# An innovative cholesteric liquid crystal biosensor enabling high-contrast colorimetric detection and haze-based quantitation

**DOI:** 10.1186/s13036-025-00603-y

**Published:** 2026-01-06

**Authors:** Tien-Hung Peng, Chia-Tung Chang, Mon-Juan Lee, Wei Lee

**Affiliations:** 1 Institute of Lighting and Energy Photonics, College of Photonics, National Yang Ming Chiao Tung University, Guiren Dist., Tainan, 711010 Taiwan; 2 Department of Biomedical Science and Technology, College of Medicine, National Sun Yat-Sen University, Gushan Dist., Kaohsiung, 804201 Taiwan; 3 Department of Chemical and Materials Engineering, National Kaohsiung University of Science and Technology, Sanmin Dist., Kaohsiung, 807618 Taiwan; 4 Department of Medical Science Industries, Chang Jung Christian University, Guiren Dist., Tainan, 711301 Taiwan; 5 Institute of Imaging and Biomedical Photonics, College of Photonics, National Yang Ming Chiao Tung University, Guiren Dist., Tainan, 711010 Taiwan

**Keywords:** Label-free biosensor, Cholesteric liquid crystal, Bovine serum albumin, Cancer biomarker, Haze, Light scattering

## Abstract

**Background:**

A cholesteric liquid crystal (CLC) system comprising the calamitic LC 8CB (4-octyl-4’-cyanobiphenyl) doped with the chiral agent R5011 was applied as a biosensing medium.

**Results:**

By exploiting the pronounced light-scattering behavior at the smectic-to-chiral nematic transition temperature of 26.5 °C of the vertically aligned CLC, a distinct red–green (signal–background) color contrast was observed in the optical texture of the CLC in the presence of the protein standard bovine serum albumin (BSA) or the cancer biomarker CA125, with the red color intensity correlated positively with the concentration of the biological analyte. The high-contrast signal–background color scheme of the 8CB/R5011 CLC was confirmed through theoretical calculations to simulate the interference spectra and the corresponding color observed under a polarizing optical microscope. The simulation results also indicate that the collective tilt angle of the CLC increased in the presence of biomolecules, suggesting that the alignment of CLC was disrupted and light scattering was enhanced. By incorporating haze measurements as the quantitative approach for the CLC-based biosensor, a strong correlation between the mean haze value and the analyte concentration was demonstrated. The limit of detection (LOD) achieved through haze analysis was 1.23 × 10⁻¹² g·mL⁻^1^ for BSA and 3.85 × 10⁻¹⁰ g·mL⁻^1^ for CA125, which were substantially lower than those obtained via conventional image analysis (2.21 × 10⁻¹¹-g·mL⁻^1^ BSA and 1.43 × 10⁻⁹-g·mL⁻^1^ CA125). In a proof-of-concept demonstration with CA125-spiked human serum as the analyte, the LOD remained unaffected by interferents present in human serum.

**Conclusions:**

The CLC-based biosensing technology combined with haze-based quantitation offers a label-free, highly sensitive, rapid response, cost-effective, and versatile platform for detecting biological analytes, manifesting significant potential for applications in early disease diagnosis and biomedical research.

**Supplementary Information:**

The online version contains supplementary material available at 10.1186/s13036-025-00603-y.

## Introduction

Since the concept of biosensors was first proposed [1], the field of biosensing technologies has witnessed remarkable and rapid development. Notably, the pioneering study conducted by Abbott et al. [2] marked a turning point, drawing widespread attention to liquid crystal (LC)-based biosensing technologies [3]. LC detection systems have emerged as a prominent and expanding area in biomedical research due to several merits, including low cost, rapid response or efficiency, and simple operation.

Traditional LC biosensors take advantage of the birefringent properties of LCs and their sensitive response to biomolecules, which alter (usually disrupt) the alignment of the molecular director and result in noticeable changes in optical texture when observed under a polarizing optical microscope (POM), thereby enabling biomolecular detection. Various LC-based biosensing technologies have been developed based on the optical properties of LCs [4], which can be integrated in diverse platforms such as microfluidic systems [5], spin-coated LC films on a single glass substrate [6], LC–aqueous interfaces [7], and LC droplets [8]. While the optical texture of LCs provides a straightforward detection strategy, this semi-quantitative method encounters challenges related to reproducibility, practical application, and quantitative capability [9]. Although relatively scarce, novel LC-based electrochemical and fluorescence sensors that employ signal outputs other than the optical response of LCs have been developed [10, 11].

Most LC-based biosensing approaches utilize the nematic LC 5CB as the transducing probe [12]. This single compound has a nematic temperature range of merely 11 °C and a low nematic phase stability, becoming isotropic at 35 °C. Over the years, our group has proposed the biosensing application of nematic LCs with higher phase stabilities, such as the eutectic mixtures E7 and HDN, which is a nematic LC of high birefringence (Δ*n* = 0.333), to ensure consistent anisotropic properties and enhance optical responses [13, 14]. The potential of lyotropic LCs, including aqueous disodium cromoglycate and sunset yellow, as biosensing media has also been explored, which demonstrated detection performance comparable to that of thermotropic LCs [15]. Cholesteric LCs (CLCs) are an intriguing type of thermotropic LCs that have been relatively less explored in the context of biomedical sensing. CLCs are formed by incorporating a chiral dopant into a nematic host, resulting in a self-organized, helically twisted structure with a defined periodicity. The helical pitch can be precisely modulated by adjusting the concentration of the chiral agent. When the pitch length matches the wavelength of incident light, CLCs exhibit selective reflection of circularly polarized light, a phenomenon known as the Bragg reflection. CLCs are highly responsive to external stimuli, such as surface anchoring, temperature fluctuations, externally applied electric fields, and light exposure, which induce CLCs to transition between various optical states, including the homeotropic (H), (Grandjean) planar, fingerprint, and focal conic (FC) states [16]. Owing to this rich tunability, CLCs have become promising materials in display technologies and smart window applications, enabling advanced functions such as reversible dual-mode haze switching [17].

In terms of biosensing technologies, several CLC-based optical detection approaches have been proposed. For biodetection at the CLC–water interface, Lee et al. developed biosensors for small-molecule metabolites such as glucose and cholesterol based on CLC droplets prepared using a microfluidics approach [18]. For biodetection at the CLC–glass interface, our group previously reported a color-indicating CLC-based biosensor for BSA using transmission spectrometry as the quantitative approach [19]. A photocontrolled capacitive biosensor based on a photoresponsive azobenzene-doped LC (ADLC), which can be induced to shift reversibly between CLC and nematic states through *trans*–*cis* photoisomerization, facilitated the multi-mode detection of BSA [20]. A simplified single-substrate biosensing platform constructed with CLC films was reported, demonstrating biosensing potential comparable to that of conventional LC cells [6]. Nevertheless, the full potential of CLCs for quantitative analysis and rapid detection remains to be explored. To address these gaps, this study proposes a novel CLC system composed of the calamitic LC 8CB (4-octyl-4’-cyanobiphenyl) doped with a right-handed chiral dopant, R5011. The application of 8CB has been reported in the field of optoelectronics [21, 22], and its physical properties, such as phase transition temperatures and birefringence, have been well documented [23, 24]. The chiral dopant R5011, commonly employed in photonic research [25], exhibits high helical twisting power (HTP), making it an ideal candidate for chiral induction in LC systems. Furthermore, molecular docking and Raman spectroscopy studies have confirmed strong affinity between 8CB and bovine serum albumin (BSA), highlighting 8CB’s potential as a cost-effective and highly sensitive biosensing material [26]. By utilizing the vertical anchoring strength of the surface alignment reagent dimethyloctadecyl[3-(trimethoxysilyl)propyl] ammonium chloride (DMOAP), as well as the polymorphic state of the 8CB/R5011 CLC in the smectic-to-chiral nematic transition at 26.5 °C, a stronger light scattering is expected to occur to amplify the optical signal induced by biomolecular interactions. In addition, to enhance the reliability and precision of biosensing, haze measurement as a “complementary” optical readout was incorporated alongside conventional texture analysis. Haze quantification provides an objective metric of light scattering through the LC layer, which correlates with molecular disruptions at the LC–analyte interface. Unlike colorimetric detection, which can be influenced by ambient lighting or subjective interpretation, haze measurement offers standardized, reproducible data that improves sensitivity and dynamic range. This dual-readout strategy strengthens the analytical robustness of the sensor and supports quantitative evaluation of biomolecular interactions.

In this study, the protein standard BSA and the ovarian cancer biomarker CA125 were applied as the analyte. BSA is commonly used in protein assays to construct calibration curves to determine the concentration of unknown protein samples. It is an α-helical, globular protein similar to many biologically relevant serum proteins, and therefore a suitable model protein for examining general sensor responses to proteins. CA125, commonly evaluated during cancer screenings, is selected as a proof-of-concept cancer biomarker for potential clinical application. This study aims to develop a clinically relevant and quantitative biosensing platform based on the 8CB/R5011 CLC by leveraging both the unique optical properties of CLC and the rapid measurement capability provided by the haze meter, paving the way for impactful applications in healthcare and diagnostics.

## Experimental

### Optical texture observation and image analysis

The materials (Fig. S1) used in this study, characterization of the sensing medium 8CB/R5011 (Figs. S2 and S3), and detailed methods for the preparation of LC cells for the detection of BSA (Fig. S4) and CA125 (Fig. S5) were described in Sections S1 and S2 of the Supplementary Information. Optical texture observation was performed on the assembled LC cell immobilized with BSA or CA125 immunocomplex using a crossed POM in the transmission mode (OLYMPUS BX51-P, Olympus Corp., Tokyo, Japan). Images were captured with a digital camera and analyzed using the open-source software ImageJ with a fixed pixel size of 1536 × 1536 pixels [27].

### Haze measurements

**Fig. 1:**
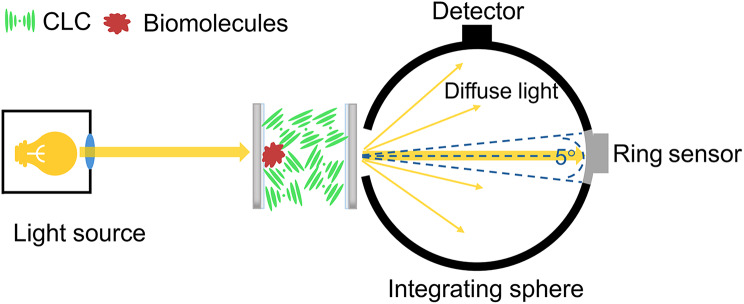
The experimental setup for the haze measurement of a CLC-based biosensor. The haze meter measures the intensity of diffuse light, or scattered light with a scattering angle larger than 2.5°, from the LC cell relative to the total transmitted light

Haze measurements were conducted using a haze meter (COH-5500, Nippon Denshoku Corp., Japan), which comprises a calibrated light source and an integrating sphere with a 0.5 × 0.5 cm detection aperture. This setup was used to evaluate the haze of confined CLC films in the presence of biological analytes, as shown in Fig. 1. Haze is defined as the percentage ratio of the intensity of diffuse light (i.e., the incident light scattered by more than 2.5 degrees), *I*_d_, to that of the total transmitted light, *I*_t_, captured by the integrating sphere, as expressed by:

1 $$\mathrm{Haze} (\% )=\left( {\frac{{{\operatorname{I} _d}}}{{{\operatorname{I} _t}}}} \right) \times 100{{\% }}$$

### Transmission measurements

Transmittance in the 400–700 nm range was measured using a fiber optic spectrometer (Ocean Optics HR2000+), which also enabled determination of the LC cell gap via visible-light interference. The distance between the sample and the sensor was around 7 cm, and the detector had a diameter of about 0.5 cm, resulting in a collection angle of approximately 4°.

Additional measurements using various instruments for material characterization, along with birefringent texture color simulations (Figs. S6 and S7, and Table S1), are detailed in Sections S1–S3 of the Supplementary Information.

## Results and discussion

### Optical texture and color simulation of the 8CB/R5011 CLC

**Fig. 2:**
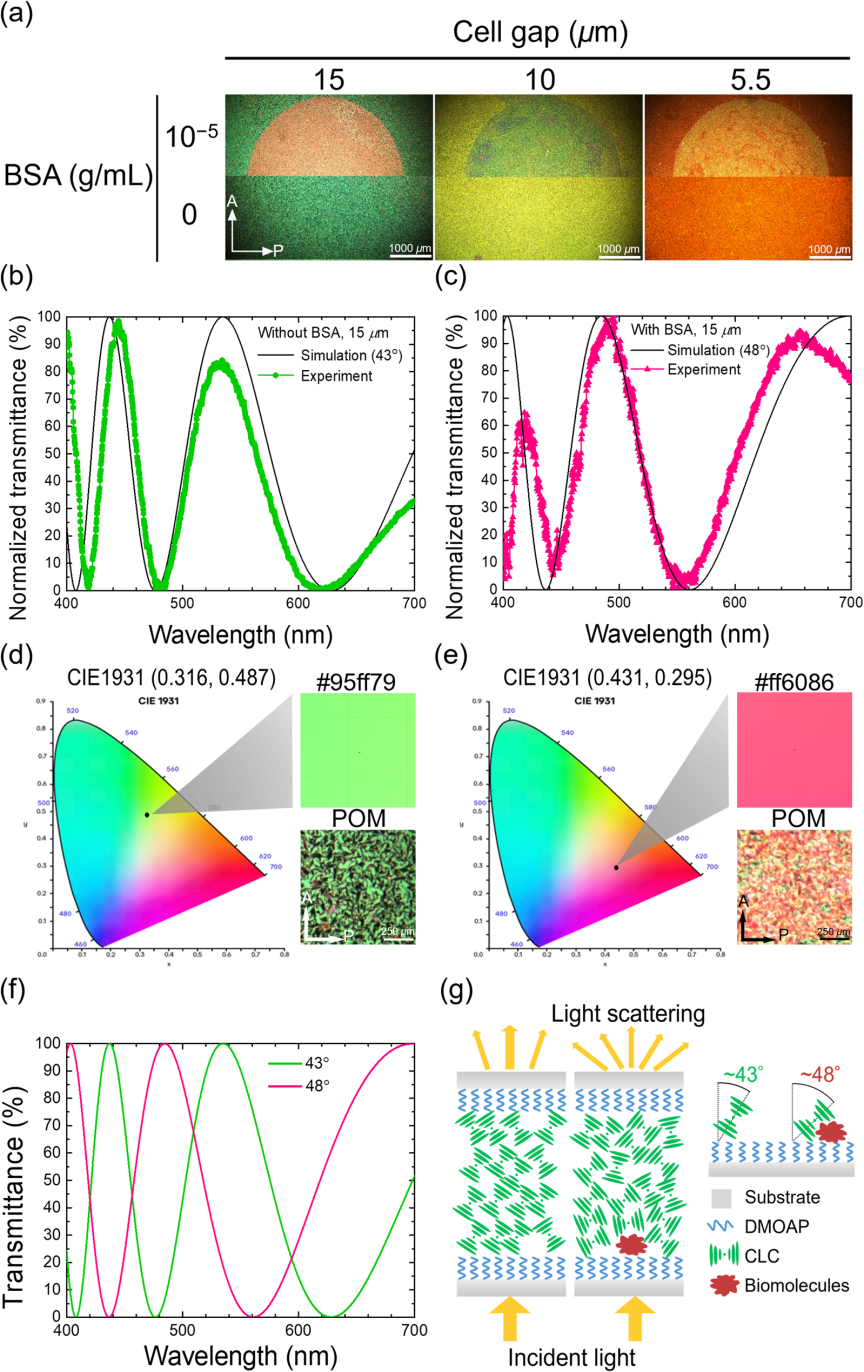
Effect of cell gap and protein adsorption on the interference color of the 8CB/R5011 CLC. (**a**) Optical textures of the 8CB/R5011 samples at different cell gaps (15, 10, and 5.5 μm) at 0-g·mL^–1^ BSA (bottom half images) and in the presence of 10^− 5^-g·mL^–1^ BSA (top half images). Simulated interference spectra of the 8CB/R5011 CLC at specific tilt angles were produced to fit the normalized experimental interference spectra in the (**b**) absence and (**c**) presence of BSA at a cell gap of 15 μm. The simulated birefringence-induced interference hues of the 8CB/R5011 CLC predicted from the CIE 1931 chromaticity coordinates were compared with that observed under the POM in the (**d**) absence and (**e**) presence of BSA at a cell gap of 15 μm. (**f**) Comparative visible interference spectra simulated at tilt angles of 43° and 48°. (**g**) Schematic of the working principle of the 8CB/R5011 CLC-based biosensor

Temperature is a dominant factor in the phase transition of CLC [28]. For 8CB doped with 2-wt% R5011, a smectic-to-chiral nematic (N*) transition occurred at 26.5 °C, and a clearing point was observed at 38 °C, as determined by temperature-dependent dielectric measurements (Fig. S3). At the phase-transition temperature of 26.5 °C, the 8CB/R5011 CLC exhibited a mixed state of smectic and chiral nematic phases, resulting in a sensing medium that is potentially more susceptible to disruption by analytes. In the absence of BSA at 26.5 °C, the optical texture under the POM with crossed polarizers appeared green for the 8CB/R5011 CLC containing 2-wt% R5011 in a LC cell of 15 μm in thickness (Fig. 2(a)). In the presence of 10⁻^5^-g·mL^–1^ BSA, the area where BSA was immobilized appeared red against a green background. When the cell gap was reduced to 10 and 5.5 μm, the optical texture of the 8CB/R5011 CLC changed from the distinctive red–green color contrast at a cell gap of 15 μm to a signal–background color combination of cyan and yellow and then to yellow and orange, respectively.

The unique color scheme of the 8CB/R5011 CLC at varying cell gaps prompted a deeper investigation into the relationship between birefringence-induced optical interference and the resulting hues observed in the optical textures of the anisotropic CLC [29]. As shown in Figs. 2(b) and (c), the simulated transmission spectra for the 8CB/R5011 CLC were generated at a cell gap of 15 μm and at specific tilt angles (defined as the angles between the effective helical axes and the substrate normal) to match the experimental spectra, according to the procedure described in Section S3 of the Supplementary Information. By comparing Figs. 2(b) and (c), the effective tilt angle of the helical axis (i.e., optic axis) of the 8CB/R5011 CLC increased from 43° to 48° at a cell gap of 15 μm in the presence of BSA. A similar increase in tilt angle, from 45° to 49° (compare Figs. S7(a) and (b)) and from 46° to 50° (compare Figs. S7(e) and (f)), was seen at a cell gap of 10 and 5.5 μm, respectively, when BSA was immobilized. Results from these theoretical calculations were subsequently mapped to the CIE 1931 chromaticity coordinates to predict the color of the optical texture in the presence or absence of BSA. The predicted color for the 8CB/R5011 CLC at a 15-µm cell thickness, as seen in Figs. 2(d) and (e), was in good agreement with the interference color observed under the POM with crossed polarizers. At cell gaps of 10 and 5.5 μm, the computed interference colors of the 8CB/R5011 CLC were also comparable to the observed POM images (Figs. S7(c)–(d) and (g)–(h)). It is hypothesized that upon interaction with BSA, the tilt angle of the 8CB/R5011 CLC increases by approximately 5°, resulting in enhanced light scattering and a blue-shift of the interference peak that alters the observed color (Figs. 2(f) and (g)). The distinct color schemes and the significant color change in the presence of BSA manifest that the 8CB/R5011 CLC can be employed as a biosensing medium. Due to the high-contrast color combination of red and green, as opposed to the cyan–yellow and yellow–orange color schemes, the cell gap of 15 μm was adopted for the LC cell assembled as the platform in the following studies. By virtue of the polymorphic nature of the 8CB/R5011 CLC at 26.5 °C, the visual color distribution is often non-uniform—especially in the presence of biomolecules—leading to slightly better agreement between simulation and experiment for biomolecule-free samples. Nonetheless, the overall hues remain consistent.

### Optimization of the biosensing conditions of the 8CB/R5011 CLC

**Fig. 3:**
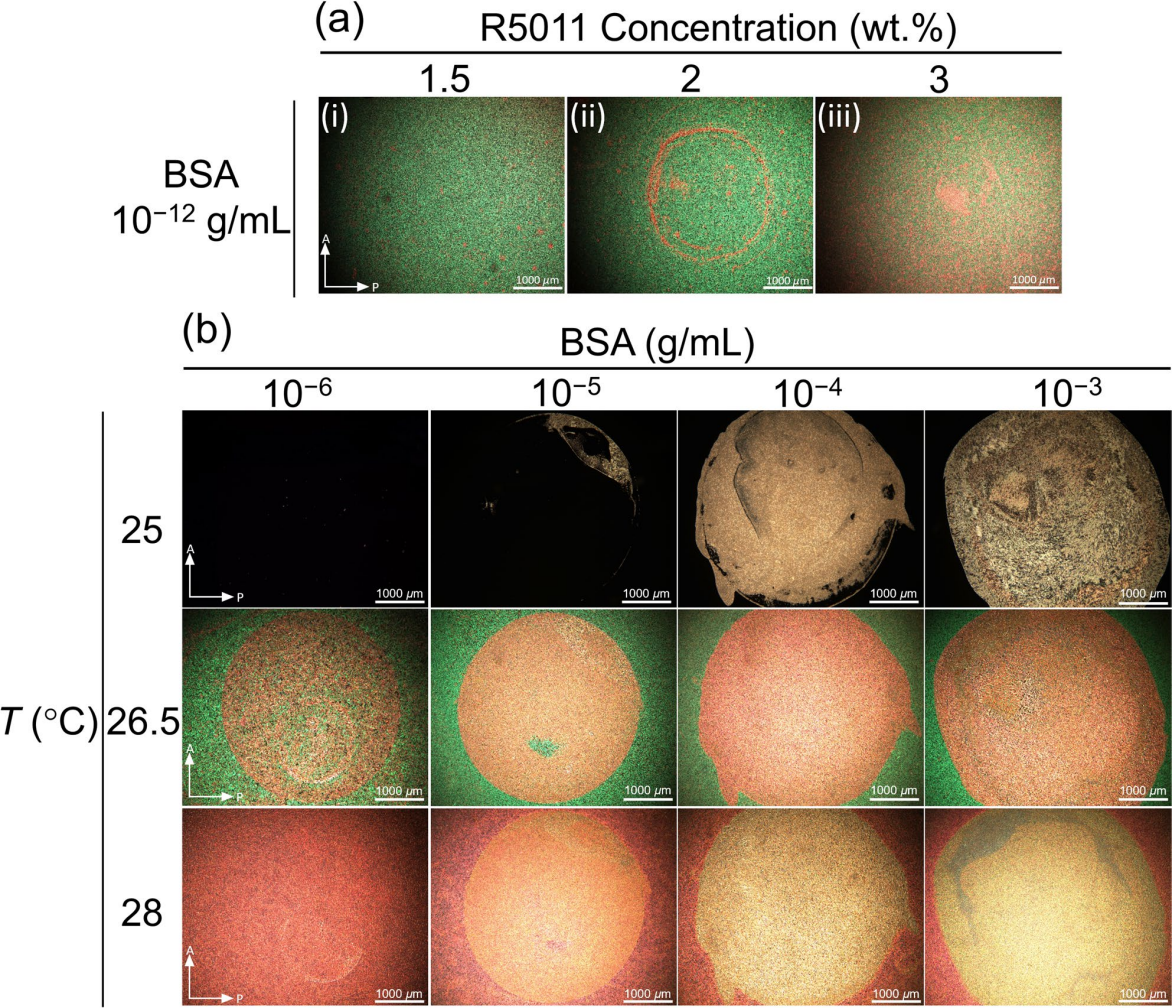
Optimization of the R5011 doping concentration and detection temperature of the 8CB/R5011 CLC-based biosensing platform. (**a**) Polarizing micrographs of the 8CB/R5011 mixture containing 1.5-, 2-, and 3-wt% R5011 in the presence of 10^−12^-g·mL^–1^ BSA. (**b**) Temperature-dependent variation in the optical texture of the 8CB/R5011 CLC with 2-wt% R5011 in the presence of 10^−6^–10^−3^-g·mL^–1^ BSA

To investigate the optimal concentration of the chiral dopant R5011 for CLC-based biosensing, three different doping concentrations, 1.5-, 2-, and 3 wt%, were employed, and the optical textures of the resulting 8CB/R5011 mixtures were observed in the presence of 10^−12^-g·mL^–1^ BSA at 26.5 °C (Fig. 3(a)). At relatively low concentrations, a droplet of protein solution tends to form a coffee ring on the DMOAP-coated glass substrate when the water evaporates, outlining the contact area of the protein solution [30]. At 10^−12^-g·mL^–1^ BSA, a red coffee-ring pattern can be clearly discerned from the green background when an 8CB/R5011 mixture containing 2-wt% R5011 was applied as the sensing medium (center panel, Fig. 3(a)). In contrast, the optical texture of the 8CB/R5011 mixture with 1.5-wt% R5011 exhibited only the green background (left panel, Fig. 3(a)), whereas that with 3-wt% R5011 gave rise to a red interference signal alongside the green background in areas without BSA (right panel, Fig. 3(a)). Consequently, 2 wt% was selected as the optimal concentration for the chiral dopant in CLC-based biosensing.

The correlation between the optical texture of the CLC and BSA concentration was next investigated at three different temperatures, 25, 26.5, and 28 °C, which represent the smectic, smectic-to-chiral nematic, and chiral nematic phases, respectively (Fig. 3(b)). At 25 °C, the 8CB/R5011 CLC was in the SmA phase with a higher order parameter (*S*), and its optical texture was completely dark when 10⁻^6^-g·mL^–1^ BSA was immobilized. This behavior can be attributed to the fact that the amount of BSA was insufficient to disrupt the alignment of the LC, resulting in a homeotropic (H) state due to the strong vertical anchoring force provided by the DMOAP alignment layers. As the BSA concentration was increased from 10⁻^5^ to 10⁻^3^ g·mL^–1^, only regions with BSA immobilization appeared red, and the red color intensity of the optical texture increased gradually with increasing amount of BSA.

When the temperature reached 26.5 °C, the 8CB/R5011 CLC rendered a red texture against the green background, and the intensity of the red component of the optical texture increased as more BSA was immobilized on the DMOAP-modified glass substrate. Optical signal resulting from BSA can still be observed at 10⁻^6^ g·mL^–1^, indicating that the limit of detection was significantly lower at 26.5 °C than at 25 °C. When the temperature was elevated further to 28 °C, the 8CB/R5011 CLC exhibited a more disordered molecular arrangement, leading to a reduced contrast between the optical signal of BSA and the background. Based on these observations, 26.5 °C was adopted as the temperature for all subsequent measurements.

### CLC-based quantitative protein assay

**Fig. 4:**
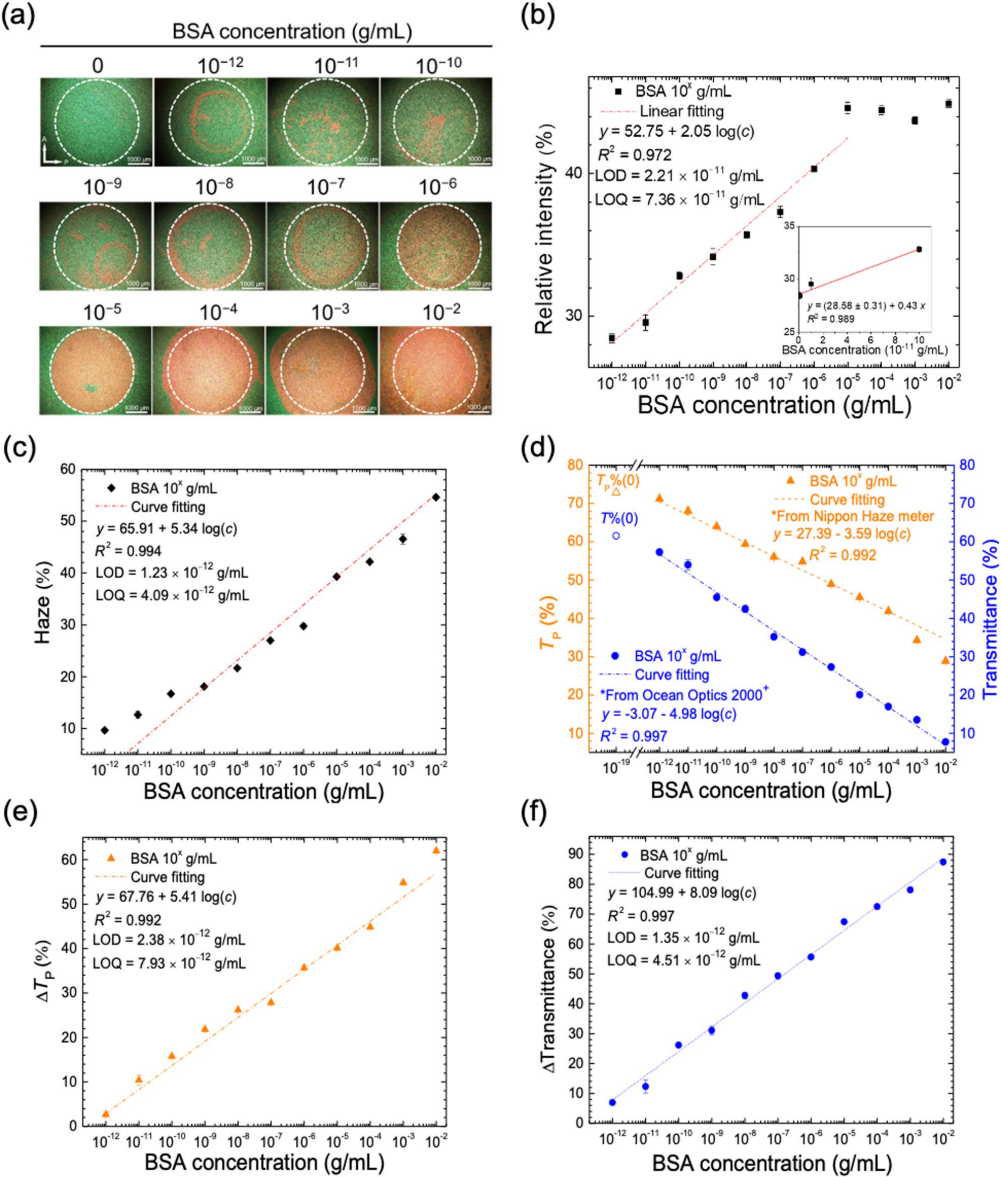
CLC-based protein assay quantitated by image analysis, haze measurement, and transmission spectrometry. (**a**) Optical textures of the 8CB/R5011 cells with BSA immobilized at various concentrations (10⁻^12^–10⁻^2^ g·mL^–1^). (**b**) Correlation between the relative intensity of the R-component (%) and BSA concentration obtained from image analysis of the optical textures in (a). Inset, relative intensity (%) plotted against BSA concentration on a linear scale in the lower-concentration region for the determination of LOD and LOQ. (**c**) Correlation between the mean haze value of the CLC and BSA concentration. (**d**) Parallel transmittance *T*_p_ and transmittance *T* acquired with a haze meter and a fiber optic spectrometer, respectively, plotted against BSA concentration. (**e**) The relative difference in *T*_p_, expressed as Δ*T*_P_ (%), calculated from (**d**) and plotted against BSA concentration. (**f**) The relative difference in *T*, expressed as Δ*T* (%), deduced from (**d**) and plotted against BSA concentration

The distinct red–green contrast resulting from BSA adhesion at 26.5 °C highlights strong potential for advanced image analysis (Fig. 3(b)). Detection of a wider range of BSA concentration was therefore performed with the 8CB/R5011 CLC for quantitative purposes. As shown in Fig. 4(a), the CLC revealed a uniform light green color in the absence of BSA. As the concentration of BSA was increased from 10⁻^12^ to 10⁻^2^ g·mL^–1^, the red color intensity of the optical texture was enhanced accordingly. Utilizing the image processing software ImageJ, the intensities of the R, G, B color channels were analyzed within a defined area, and the percent contribution of the red channel relative to the total light intensity across the RGB spectrum was calculated by the following mathematical expression:

2 $$\mathrm{R}{\mathrm{-}}\mathrm{c}\mathrm{o}\mathrm{m}\mathrm{p}\mathrm{o}\mathrm{n}\mathrm{e}\mathrm{n}\text{t } \mathrm{r}\mathrm{e}\mathrm{l}\mathrm{a}\mathrm{t}\mathrm{i}\mathrm{v}\text{e } \mathrm{i}\mathrm{n}\mathrm{t}\mathrm{e}\mathrm{n}\mathrm{s}\mathrm{i}\mathrm{t}\mathrm{y} (\% )=\left( {\frac{R}{{R+G+B}}} \right) \times 100\%, $$

where *R*, *G*, and *B* represent the absolute intensities of the red, green, and blue color channels detected by Image J, respectively. As depicted in Fig. 4(b), the relative intensity of the R-component increased from approximately 29% at 10⁻^12^-g·mL^–1^ BSA to 45% at 10⁻^5^-g·mL⁻^1^ BSA. However, when the BSA concentration exceeded 10⁻^5^ g·mL^–1^, the relative intensity leveled off and reached saturation. Regression analysis of the relative intensity-versus-concentration semi-logarithmic plot in Fig. 4(b) between 10⁻^12^ and 10⁻^5^ g·mL^–1^ of BSA yielded a calibration curve with a coefficient of determination (*R*²) of 0.972, indicating a strong positive correlation. To determine the limit of detection (LOD) and limit of quantification (LOQ), another relative intensity-versus-concentration plot was produced on a linear scale in the lower-concentration region of Fig. 4(b), as shown in the inset, followed by linear regression analysis. Within the linear calibration range, the LOD and LOQ for BSA were calculated to be 2.21 × 10⁻^11^ and 7.36 × 10⁻^11^ g·mL^–1^, respectively, in accordance with

3 $${\mathrm{LOD}}=\frac{{3s}}{m}$$

and

4 $${\mathrm{LOQ}}=\frac{{10s}}{m},$$

where *s* and *m* represent the standard deviation of the *y*-intercept and the slope of the linear regression line, respectively.

To explore other quantitative approaches for CLC-based biosensing, haze measurements were employed to study the correlation between the haze value of the 8CB/R5011 CLC and BSA concentration ranging from 10⁻^12^ to 10⁻^2^ g·mL^–1^. As can be seen in Fig. 4(c), the mean haze value of the CLC increased in a concentration-dependent manner, with the *R*² value reaching 0.994. The LOD and LOQ calculated by Eqs. (3) and (4) were 1.23 × 10⁻^12^ and 4.09 × 10⁻^12^ g·mL^–1^, respectively, which were an order of magnitude lower than those determined by image analysis of the optical texture (Fig. 4(b)). Because the relative intensity and haze value of the 8CB/R5011 CLC were positively correlated to BSA concentration, it is suggested that as more biomolecules adhere to the DMOAP-coated glass substrate, the disruption in the helical configuration of CLC molecules amplifies, bringing about more substantial light scattering. Moreover, the linear range of detection, from 10⁻^12^ to 10⁻^2^ g·mL^–1^ for BSA (Fig. 4(c)), was wider than that obtained from image analysis of the optical texture, which was from 10⁻^12^ to 10⁻^5^ g·mL^–1^ (Fig. 4(b)). Note that detection sensitivity of the haze-based quantitative approach was also improved, as the slope of the linear regression line in Fig. 4(c) was significantly larger than that in Fig. 4(b). The promoted detection sensitivity and improved LOD achieved when employing haze measurement for quantitation may stem from the more accurate measurement of scattered light in the integrating sphere of the haze meter, which collects scattered light three-dimensionally in all directions and may therefore contribute to a wider dynamic range (the range between the smallest and the largest signal without saturation). In contrast, quantitation through image analysis is based on a fixed two-dimensional area of interest with a rather limited dynamic range, where the color intensity reaches saturation within a smaller range of detection.

Additionally, parallel transmittance *T*_P_, measured by the haze meter within a light collection angle of ± 2.5°, can also be applied in protein quantitation. Results from *T*_P_ measurements were compared with white-light transmittance *T* measured with a conventional fiber optic spectrometer to validate the feasibility of applying *T*_p_ as a supplementary parameter for quantitation. In Fig. 4(d), both *T*_P_ and *T* decreased as BSA concentration was increased from 10⁻^12^ to 10⁻^2^ g·mL^–1^, with *T*_P_ declining from approximately 70% to 30% and *T* dropping from 60% to 7%. The value of *T*_P_ was consistently higher than *T* at all tested concentrations of BSA. This discrepancy can be attributed to the wider light collection angle of the haze meter (5°), which allows it to capture more light. In comparison, the fiber optic spectrometer has a light collection angle of approximately 4°. Linear regression analysis indicates that both plots in Fig. 4(d) had *R*² values over 0.99, but the absolute value of the slope was higher for the plot of *T* versus BSA concentration, pointing to a potentially higher detection sensitivity. Nevertheless, these results provide evidence for the reliability of using *T*_P_ acquired with the haze meter to quantitate the amount of protein.

The negative correlation as shown in Fig. 4(d) can be converted to a positive one by

5 $$\Delta T(c)\% =\frac{{T(0) - T(c)}}{{T(0)}} \times 100\%, $$

where *T* (0) represents the transmittance in the absence of BSA, and *T* (*c*) refers to that at a given analyte concentration *c*. It transforms measured (parallel) transmittance into the relative difference in *T*_P_ or *T*, which is expressed as Δ*T*_P_ (%) or Δ*T* (%), respectively. As illustrated in Figs. 4(e) and (f), Δ*T*_P_ (%) and Δ*T* (%) increased with increasing BSA concentration, with a maximum relative difference of roughly 60% and 80% for Δ*T*_P_ (%) and Δ*T* (%), respectively. The LOD and LOQ determined by the plot of Δ*T*_P_ (%) versus BSA concentration were 2.38 × 10⁻^12^- and 7.93 × 10⁻^12^- g·mL^–1^ BSA, respectively, while those determined by the plot of Δ*T* (%) versus BSA concentration were 1.35 × 10⁻^12^ and 4.51 × 10⁻^12^ g·mL^–1^, respectively. These LOD and LOQ values obtained from spectrometric analysis were comparable to those derived from haze measurements (Fig. 4(c)). Through the comparison with image analysis and transmission spectrometry, this study established that the haze value and parallel transmittance recorded by the haze meter are effective quantitative parameters for the CLC-based protein assay. Furthermore, the LOD values achieved with the 8CB/R5011 CLC in this study are several orders of magnitude lower than those of most thermotropic liquid crystal-based biosensors [31], as well as colorimetric, fluorometric, and electrochemical protein assays (Table S2).

### CLC-based quantitative CA125 immunoassay

To demonstrate the potential of the CLC-based biosensing technique in a clinical setting, an immunoassay was developed for the cancer biomarker CA125. Anti-CA125 antibodies were immobilized on the DMOAP-coated glass substrate as the bioreceptor for CA125. The biosensing platform was designed by controlling the concentration of the capture antibody so that, in the presence of the anti-CA125 antibody, only the green background color of the 8CB/R5011 CLC was observed under the polarizing optical microscope. This ensures that the red optical signal observed in the presence of CA125 was indeed caused by the specific binding between the antigen and antibody. As shown in Fig. S8(a), the immobilization concentration of the anti-CA125 antibody was optimized using three different concentrations, 10⁻^11^, 10⁻^10^, and 10⁻^9^ g·mL⁻^1^. The red color intensity in the optical texture of the 8CB/R5011 CLC was enhanced when the concentration of the anti-CA125 antibody was increased, prompting a coffee ring at 10⁻^9^ g·mL⁻^1^. The optical signal caused by the anti-CA125 antibody was indiscernible from the background when its concentration was 10⁻^10^ g·mL⁻^1^ or lower. The threshold value of 10⁻^10^-g·mL⁻^1^ anti-CA125 antibody, with a background haze value of 13% (Fig. S8(b)), was therefore selected as the optimized concentration for antibody immobilization to avoid false-positive signals in the absence of CA125. To further minimize non-specific binding, the glass substrate was treated with ethanolamine (ETA) after antibody immobilization to prevent protein adsorption on areas without anti-CA125 antibodies [32, 33]. When 10⁻^10^-g·mL⁻^1^, immobilized anti-CA125 antibody was reacted with 10⁻^5^-g·mL⁻^1^ CA125, non-specific binding caused by excess CA125 was significantly reduced in the area without antibody immobilization after ETA modification (Fig. S8(c)).

**Fig. 5:**
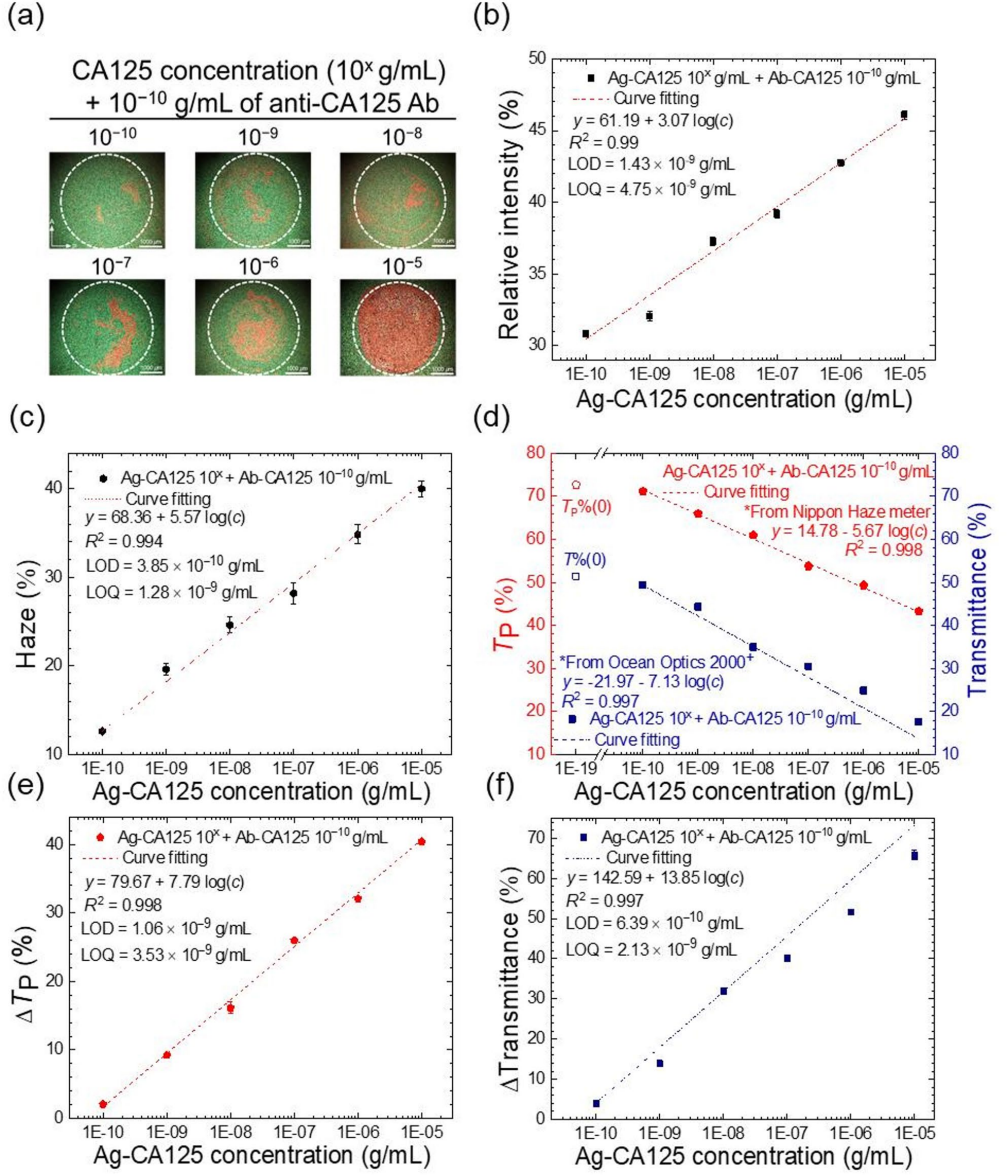
CLC-based CA125 immunoassay quantitated by image analysis, haze measurement, and transmission spectrometry. (**a**) Optical textures of the 8CB/R5011 cells with 10⁻^10^–10⁻^5^-g·mL^–1^ CA125 reacting with 10⁻^10^-g·mL^–1^, immobilized anti-CA125 antibody. (**b**) Correlation between the relative intensity of the R-component (%) and CA125 concentration obtained from image analysis of the optical textures in (**a**). (**c**) Correlation between the mean haze value of the CLC and CA125 concentration. (**d**) Parallel transmittance *T*_p_ and transmittance *T* measured by a haze meter and a fiber optic spectrometer, respectively, plotted against CA125 concentration. (**e**) The relative difference in *T*_p_, expressed as Δ*T*_P_ (%), calculated from (**d**) and plotted against CA125 concentration. (**f**) The relative difference in *T*, expressed as Δ*T* (%), calculated from (**d**) and plotted against CA125 concentration

The CLC-based CA125 immunoassay was conducted at concentrations spanning from 10⁻^10^ to 10⁻^5^ g·mL^–1^ of CA125 under the optimized conditions described above. As presented in Fig. 5(a), the optical texture of the 8CB/R5011 CLC in the presence of CA125 exhibited a red–green color contrast similar to that observed with BSA, where the red optical signal indicating the formation of the CA125 immunocomplex intensified with increasing CA125 concentration. Results from image analysis revealed that the average relative intensity of the R-component increased from 30% to 47% as CA125 concentration was increased from 10⁻^10^ to 10⁻^5^ g·mL^–1^ (Fig. 5(b)). Notably, at CA125 concentrations exceeding 10⁻^5^ g·mL^–1^, the relative intensity of the R-component plateaued and did not increase further (data not shown); therefore, the range of detection (ROD) for the CLC-based immunoassay was defined as 10⁻^10^ to 10⁻^5^ g·mL^–1^ for CA125. The regression line in Fig. 5(b) had an *R*² value of 0.99, and an LOD of 1.43 × 10⁻^9^ g·mL^–1^ and LOQ of 4.75 × 10⁻^9^ g·mL^–1^ CA125 was obtained. On the other hand, the binding specificity of the CLC-based immunoassay was demonstrated by reacting 10⁻^10^-g·mL⁻^1^, immobilized anti-CA125 antibody with 10⁻^5^-g·mL⁻^1^ BSA (Fig. S8(d)). The optical texture of the 8CB/R5011 CLC in the presence of BSA remained similar to that observed when only the anti-CA125 antibody was immobilized, implying that BSA did not bind to the anti-CA125 antibody, and the red optical signal observed in Fig. 5(a) arose from the specific binding between CA125 and the anti-CA125 antibody.

In the haze-based quantitative analysis, improvements in LOD and LOQ, along with enhanced detection sensitivity, were noted when compared with the results from image analysis of the CLC optical texture, similar to the CLC-based protein (BSA) assay in Fig. 4. The average haze value of the 8CB/R5011 CLC increased from 10% to 40% as the CA125 concentration rose from 10⁻^10^ to 10⁻^5^ g·mL^–1^ (Fig. 5(c)). The regression line in Fig. 5(c) had an *R*² value of 0.994, indicating that changes in CA125 concentration can be clearly reflected by haze measurements. In addition, the slope of the linear regression line in Fig. 5(c) was nearly twice that obtained via image analysis (Fig. 5(b)), and the LOD and LOQ values were further reduced to 3.85 × 10⁻^10^ and 1.28 × 10⁻^9^ g·mL^–1^, respectively, an order of magnitude lower than those determined by image analysis.

The transmittance, *T*_P_ (%) and *T* (%), of the CLC at various concentrations of CA125 was next measured using a haze meter and a fiber optic spectrometer, respectively. As delineated in Fig. 5(d), the measured values of *T*_P_ (%) and *T* (%) decreased from 70% to 40% and from 50% to 20%, respectively, with increasing CA125 concentration from 10⁻^10^ to 10⁻^5^ g·mL^–1^. Both regression lines in Fig. 5(d) had *R*² values exceeding 0.99, with the plot of *T* (%) versus concentration displaying a larger slope, indicating higher detection sensitivity. The results shown in Fig. 5(d) were converted to relative differences, Δ*T*_P_ (%) and Δ*T* (%), as depicted in Figs. 5(e) and (f), respectively, according to Eq. (5). LOD and LOQ were calculated to be 1.06 × 10⁻^9^ and 3.53 × 10⁻^9^ g·mL^–1^, respectively, from the plot of Δ*T*_P_ (%) versus CA125 concentration. Additionally, the LOD and LOQ values were found to be 6.39 × 10⁻^10^ and 2.13 × 10⁻^9^ g·mL^–1^, respectively, when determined from the plot of Δ*T* (%) versus CA125 concentration.

**Fig. 6:**
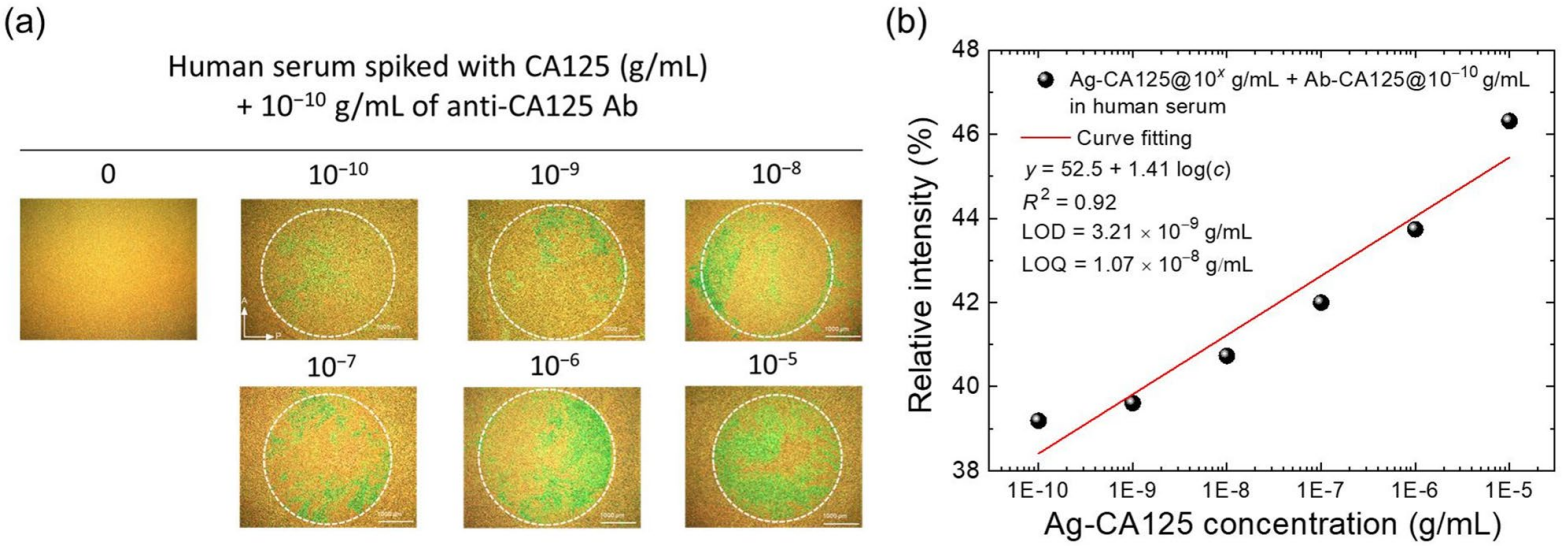
CLC-based CA125 immunoassay of diluted human serum spiked with various concentrations of CA125. (**a**) Optical textures of the 8CB/R5011 cells with 1:100-diluted human serum spiked with 10⁻^10^–10⁻^5^-g·mL^–1^ CA125 reacting with 10⁻^10^-g·mL^–1^, immobilized anti-CA125 antibody. (**b**) Correlation between the relative intensity of the G-component (%) and CA125 concentration obtained from image analysis of the optical textures in (**a**)

To strengthen specificity validation and evaluate the matrix effect, the 8CB/R5011 CLC-based CA125 immunoassay was performed using 1:100-diluted human serum spiked with CA125 as the analyte. Due to the high concentration of proteins, electrolytes, hormones, nutrients, and other amphiphiles in human serum, our usual procedure for rinsing the glass substrate was insufficient for eliminating nonspecific binding of interferents from human serum. As shown in Fig. 6(a), a relatively large amount of serum components adsorbed nonspecifically to the DMOAP-coated glass substrate immobilized with the anti-CA125 antibody, leading to a red shift of the background color of the 8CB/R5011 CLC from green to yellow–orange. In the presence of CA125, the region with antibody immobilization reverted to green, and the relative intensity of the green component in the POM image increased with higher concentration of CA125. This is consistent with the significantly stronger affinity of CA125 for the immobilized anti-CA125 antibody compared to nonspecific serum components, leading to the displacement of loosely bound serum interferents. Results presented in Fig. 6(a) were similar to our previous observation with a high-birefringence nematic LC, in which human serum gave rise to a birefringent background signal, but the presence of CA125 resulted in a concentration-dependent increase in the relative intensity of the optical signal in the area immobilized with anti-CA125 antibodies [34].

The shift in the signal–background color combination of the 8CB/R5011 CLC observed under crossed polarizers arises from changes in phase retardation or variations in optical path length, which is determined by the product of the refractive index and the physical thickness. As seen in Fig. 2(a), differences in cell gap led to distinct color sets for both the background and signal in the optical texture. In the detection of the CA125-spiked serum samples, the altered color set can be readily explained by the additional phase contribution from nonspecific adsorption onto the DMOAP-coated glass substrate. On the other hand, results from water contact angle measurements indicate that the formation of the antigen–antibody immunocomplex enhances surface hydrophilicity (Fig. S9), confirming the presence of both antigen and antibody on the glass surface, thereby changing the interfacial interaction at the LC–glass interface and perturbing the CLC tilt angle. For the purpose of quantitative analysis under a different color scheme in the presence of human serum, the percent relative intensity of the green component in the POM image was calculated and plotted against CA125 concentration (Fig. 6(b)). Results from curve fitting produced an *R*² value of 0.92, suggesting that the green component in the POM image accurately represented the variations in CA125 concentration. In addition, an LOD of 3.21 × 10⁻^9^-g·mL^–1^ CA125 was determined through linear regression, which was of the same order of magnitude as that in the control environment (Fig. 5(b)). This indicates that the performance of the 8CB/R5011 CLC-based biosensor remained unaffected by the interference of human serum. When comparing ROD and LOD of this study with those of other CA125 immunoassay techniques reported in the literature, the performance of the 8CB/R5011 CLC biosensor was comparable or superior to fluorescence label-based and electrochemical biosensors (Table 1). Further improvements can be achieved by adjusting the concentration or changing the type of the blocking reagent, as well as modifying the rinsing procedure to lower the background signal derived from human serum.

**Table 1:** Comparison of the range of detection (ROD) and the limit of detection (LOD) of CA125 immunodetection techniques

CA125 immunodetection techniques	ROD (g·mL^–1^)	LOD (g·mL^–1^)	References
Single-substrate detection with spin-coated LC film	10^− 8^–10^− 4^	10^− 8^	[6]
Electrochemical immunosensor with Au/Pt nanostructured screen-printed carbon electrodes	3 × 10^− 6^–0.45	0.38 × 10^− 6^	[35]
Screen-printed graphene electrochemical biosensor	0.9 × 10^− 9^– 0.2 × 10^− 4^	0.92 × 10^− 6^	[36]
Immunodetection by LCs with large birefringence	10^− 10^–10^− 6^	10^− 10^	[37]
Fluorescence detection based on aptamer-functionalized silver and silver/gold nanoclusters	2–6700 × 10^− 9^	1.26 × 10^− 9^	[38]
Nematic DSCG-based optical immunosensor	10^− 12^–10^− 5^	1.92 × 10^− 10^	[39]
Electrochemical immunosensor based on ZnO nanorods‒Au nanoparticles nanohybrids	2.5 × 10^− 6^–1	2.5 × 10^− 6^	[40]
Immunosensor based on poly-anthranilic acid modified screen-printed electrodes	2.5–12.5 × 10^− 9^	1.0 × 10^− 9^	[41]
Nematic SSY-based optical immunosensor	10^− 11^–10^− 7^	1.9 × 10^− 9^	[31]
CLC-based immunosensor quantitated by image analysis	10^− 10^–10^− 5^	1.43 × 10^− 9^ (DI water) 3.21 × 10^− 9^ (serum)	Current work
CLC-based immunosensor quantitated by haze measurement	10^− 10^–10^− 5^	3.85 × 10^− 10^ (based on haze) 1.06 × 10^− 9^ (based on Δ*T*_p_)	Current work
CLC-based immunosensor quantitated by transmission spectrometry	10^− 10^–10^− 5^	6.39 × 10^− 10^ (based on Δ*T*)	Current work

## Conclusion

This study presents a novel 8CB/R5011 CLC-based biosensor exhibiting a distinct signal–background color contrast, with BSA or CA125 giving rise to a red optical signal against a green background when observed under the POM at the smectic-to-chiral nematic transition temperature of 26.5 °C in a LC cell with a cell gap of 15 μm. Quantitative protein assay and CA125 immunoassay based on the 8CB/R5011 CLC was demonstrated with three quantitative approaches, including image analysis of the optical texture, haze measurement, and transmission spectrometry using a haze meter and a conventional spectrometer. Our findings suggest that higher detection sensitivity, lower LOD, and wider ROD can be achieved by haze-based quantitation compared to image analysis. Additionally, the performance of the 8CB/R5011 CLC-based CA125 immunoassay remained unaffected by interferents present in human serum. The high contrast red–green color combination of the 8CB/R5011 CLC was consistent with the predicted interference color derived from the simulated birefringence-induced interference spectra, which suggests that the tilt angle of the CLC increased in the presence of a biological analyte, leading to enhanced light scattering characterized by haze data. The color simulation procedure can, in turn, be used to predict and interpret the optical texture colors arising from the intrinsic optical properties of the CLC under different biosensing conditions, such as the cell gap, dopant concentration, and temperature. Appropriate adjustment of these parameters enables the generation of alternative color schemes beyond the conventional red–green contrast, thereby improving accessibility for observers with red–green color vision deficiency. In conclusion, a highly sensitive, label-free, and multi-modal sensing platform was established in this study for rapid and straightforward detection and quantitation of biological analytes by harnessing the unique optical properties of the 8CB/R5011 CLC, further advancing LC-based biosensing technologies for clinical applications.

## Supplementary Information

Below is the link to the electronic supplementary material.

Supplementary Material 1

## Data Availability

All data generated or analyzed during this study are included in the manuscript and the supplementary information.
