# Supplementary material for: An innovative cholesteric liquid crystal biosensor enabling high-contrast colorimetric detection and haze-based quantitation
Source: J Biol Eng. 2026 Jan 6;20:26. doi: 10.1186/s13036-025-00603-y (PMC12870449; doi:10.1186/s13036-025-00603-y)
Supplement: Supplementary file 1 — Supplementary Material 1 [file 13036_2025_603_MOESM1_ESM.docx]

Supplementary Information

**An innovative cholesteric liquid crystal biosensor enabling high-contrast colorimetric detection and haze-based quantitation**

Tien-Hung Peng ^a^, Chia-Tung Chang ^a^, Mon-Juan Lee ^b,c,d^ *, Wei Lee ^e^, **

^a^ *Institute of Lighting and Energy Photonics, College of Photonics, National Yang Ming Chiao Tung University, Guiren Dist., Tainan 711010, Taiwan*

^b^ *Department of Biomedical Science and Technology, College of Medicine, National Sun Yat-sen University, Gushan Dist., Kaohsiung 804201, Taiwan.*

^c^ *Department of Chemical and Materials Engineering, National Kaohsiung University of Science and Technology, Sanmin Dist., Kaohsiung 807618, Taiwan*

^d^ *Department of Medical Science Industries, Chang Jung Christian University, Guiren Dist., Tainan 711301, Taiwan*

^e^ *Institute of Imaging and Biomedical Photonics, College of Photonics, National Yang Ming Chiao Tung University, Guiren Dist., Tainan 711010, Taiwan*

*Keywords:* Label-free biosensor; Cholesteric liquid crystal; Bovine serum albumin; Cancer biomarker; Haze; Light scattering

* Corresponding author. Department of Biomedical Science and Technology, College of Medicine, National Sun Yat-sen University, Gushan Dist., Kaohsiung 804201, Taiwan.

** Corresponding author. Institute of Imaging and Biomedical Photonics, College of Photonics, National Yang Ming Chiao Tung University, Guiren Dist., Tainan 711010, Taiwan.

*E-mail addresses:* [*mjlee@mail.nsysu.edu.tw*](mailto:mjlee@mail.nsysu.edu.tw) *(M.-J. Lee), Wei.Lee@nycu.edu.tw (W. Lee).*

**S1. Materials and characterization of the 8CB/R5011 CLC**

**S2. Preparation of the CLC-based biosensing platform**

**S3. Simulations of birefringence-induced interference spectra and color generation in cross-polarized transmission through CLC textures**

**S4. Optimization of antibody concentration and demonstration of binding specificity**

**S1. Materials and characterization of the 8CB/R5011 CLC**

*Materials*

The cholesteric liquid crystal (CLC)-based biosensing platform was fabricated as a sandwich-like liquid crystal (LC) cell composed of two optical-grade glass substrates of 22.0 × 18.0 × 1.1 mm in dimensions supplied by Ruilong Glass (Miaoli, Taiwan). The arithmetical mean surface roughness (*S*_a_) of the optical-grade glass substrate was 2.6273 nm, which was measured with Sensofar® according to ISO 25178 standards. The host thermotropic LC 4’-*n*-octyl-4-cyano-biphenyl (8CB), possessing optical anisotropy ∆*n* ≅ 0.18 at the wavelength of 589.3 nm and temperature of 26.5 °C, was sourced from Alfa Aesar (Haverhill, MA, USA), with its chemical structure shown in Fig. S1(a). The chiral dopant R5011 (Fig. S1(b)), exhibiting a helical twisting power (HTP) of ~ 108 μm^−1^ in 8CB, was obtained from HCCH (Nanjing, China). Ethanolamine (ETA), employed as a biological blocking agent at a concentration of 1 M [[1](#_ENREF_1), [2](#_ENREF_2)], and the silane surfactant dimethyloctadecyl[3-(trimethoxysilyl)propyl] ammonium chloride (DMOAP) as well as the standard protein bovine serum albumin (BSA), were all obtained from Sigma-Aldrich (St. Louis, MO, USA). Recombinant human CA125/MUC16 protein and anti-CA125 antibodies were obtained from R&D Systems, Inc. (Minneapolis, MN, USA) and Santa Cruz Biotechnology, Inc. (Dallas, TX, USA), respectively. Prior to experiments, BSA and human CA125/MUC16 proteins were diluted to the desired concentrations using sterilized deionized water (DIW). To prepare CA125-spiked serum samples, human serum (Valley Biomedical, Winchester, VA, USA) was diluted 1:100 in DIW and used as the diluent.


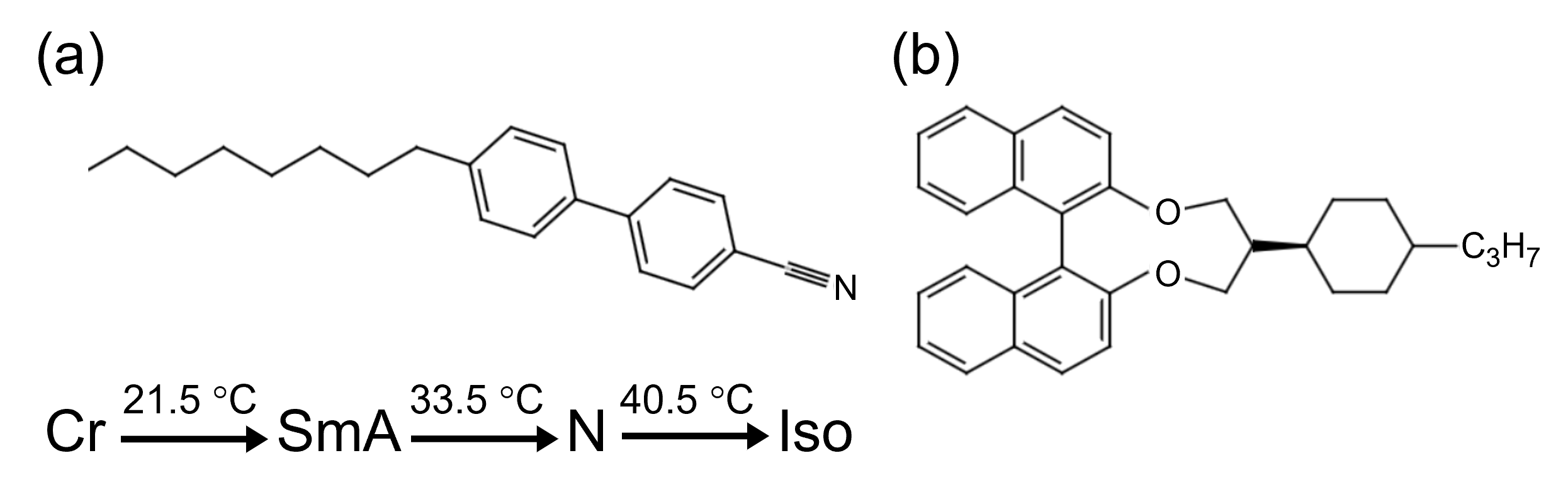


**Fig. S1.** Chemical structures of the host LC and the chiral dopant used in this study to prepare CLC. (a) The chemical structure of the host LC 8CB and its phase transition temperatures. Cr, crystal phase; SmA, smectic-A phase; N, nematic phase; Iso, isotropic phase. (b) The chemical structure of the right-handed chiral dopant R5011.

*Characterization of the 8CB/R5011 CLC*

As determined by transmission spectrometry, the CLC formed by doping 8CB with 2-wt.% R5011 had a central Bragg reflection wavelength *λ*_c_ of 744 nm (Fig. S2). This enabled the calculation of the helical pitch *P* of the 8CB/R5011 CLC using the following equation:

$\lambda_{c}=\bar{n}P\cos\theta$,

where *θ* represents the angle between the incident light and the helical axis of the CLC molecules, *n*_o_ and *n*_e_ are the ordinary and extraordinary refractive indices, respectively, and $\bar{\text{n}}$ is the average refractive index of 8CB (i.e., $\bar{n}\equiv\frac{n_{\text{e}}\text{+}n_{\text{o}}}{2}$). The HTP value of the 8CB/R5011 CLC can then be obtained as

$HTP=\left(c\cdot P\right)^{-1}$,

where *c* is the chiral dopant concentration in weight percentage. By applying Eqs. (S1) and (S2), the HTP value of R5011 in 8CB is approximately 108 μm⁻^1^.

**
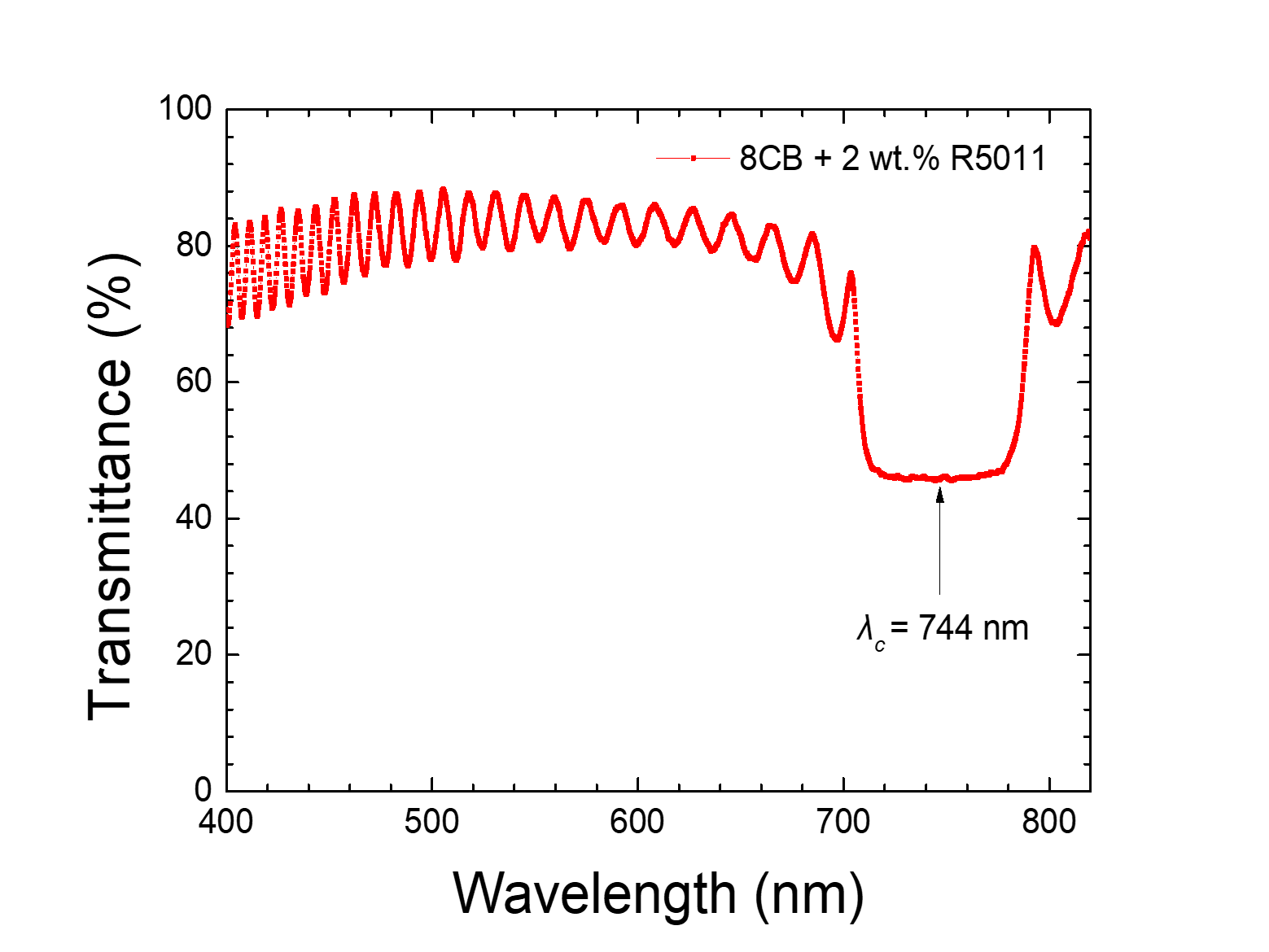
**

**Fig. S2.** Transmission spectrum (400–820 nm) of 8CB doped with 2-wt.% R5011 featuring the central Bragg reflection wavelength as indicated.

To characterize the phase transition of the CLC, a planarly aligned cell having a cell gap of 6 μm (Chiptek Corp., Miaoli, Taiwan) was filled with 8CB doped with 2-wt.% R5011, followed by dielectric measurements conducted with an Agilent E4980A high-precision LCR meter with an AC voltage of 0.5 V_rms_ as a probe over a temperature range of 20–40 °C and a frequency range of 20 Hz–200 kHz, coordinated by the graphic control program LabVIEW through a GPIB interface. The LC cell was cooled from 40 °C to 20 °C, with a thermal stabilization time of 2 minutes and a cooling rate of 0.5 °C per second. The plot of the first derivative of the real part of the dielectric constant (*ε’*) at 10 kHz with respect to temperature revealed two distinct phase transitions: a smectic-to-chiral nematic transition at 26.5 °C and a clearing point at 38 °C (Fig. S3 (a)), corresponding to the characteristic oily-streak defects associated with the Grandjean planar state of CLC at 26.5 °C and a dark texture observed at 38 °C under an OLYMPUS BX51-P polarizing optical microscope (POM), respectively (Fig. S3(b)).

**
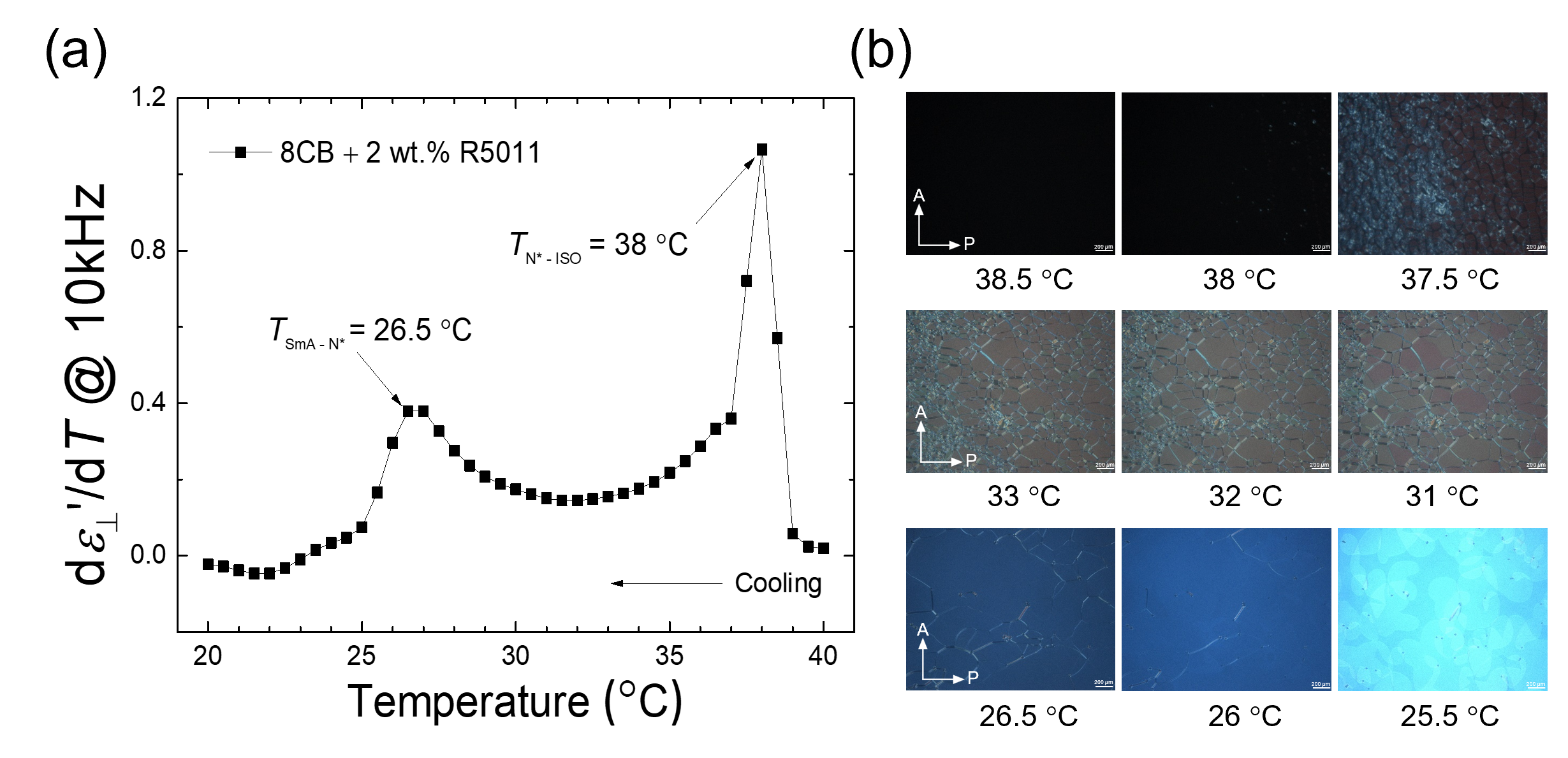
**

**Fig. S3.** Phase transitions of the 8CB/R5011 mixture. (a) Temperature-dependent variation of the first derivative of the real part of the dielectric constant for 8CB doped with 2-wt.% R5011 during the cooling process from 40 to 20 °C. (b) POM optical textures corresponding to the cooling process within the temperature range of the chiral nematic (N*) phase observed in (a).

**S2. Preparation of the CLC-based biosensing platform**

*DMOAP modification of the glass substrates*

Optical glass substrates were first cleaned by sonicating sequentially in a detergent solution for 15 minutes, followed by two additional 15-minute sonication sessions in DIW and a final 15-minute sonication in ethanol. The substrates were then dried with a stream of nitrogen and dip-coated in an ultrasonic bath containing a 1-wt.% aqueous solution of DMOAP for 30 minutes. Subsequently, the substrates were immersed in DIW and sonicated for 2 minutes to remove excess DMOAP. After drying again under nitrogen, the glass substrates were baked at 85 °C for 30 minutes to enhance the stability of the self-assembled monolayers.

*Preparation of CLC cells for BSA detection*

The complete procedure for constructing the LC cell for BSA detection is depicted in Fig. S4. A pair of DMOAP-coated optical glass substrates were used in assembling a LC cell. BSA was immobilized on the lower substrate by repeatedly dispensing 10-μL BSA solution per spot to form a 2 × 2 array (Fig. S4(c)). The substrate was then heated at 30 °C for 30 minutes on a hot plate and rinsed with DIW to remove excess biomolecules (Fig. S4(d)). A sandwich-like CLC cell was fabricated by blending rod-shaped spacers (15 μm in diameter) uniformly with AB glue and applying the resulting mixture to both opposite edges of the lower DMOAP-coated substrate immobilized with BSA (Fig. S4(f)). Another DMOAP-modified upper substrate without BSA was placed on top of the lower substrate to complete this sandwich structure (Fig. S4(g)). After drying for at least 5 minutes, the cell gap of the LC cell was determined by optical interferometry using an Ocean Optics HR2000+ high-resolution, USB fiber-optic spectrometer to ensure that it fell within the range of 15 ± 0.5 μm. Finally, the cells were filled with 6 μL of 8CB containing 2-wt.% R5011 through capillary action (Fig. S4(h)).


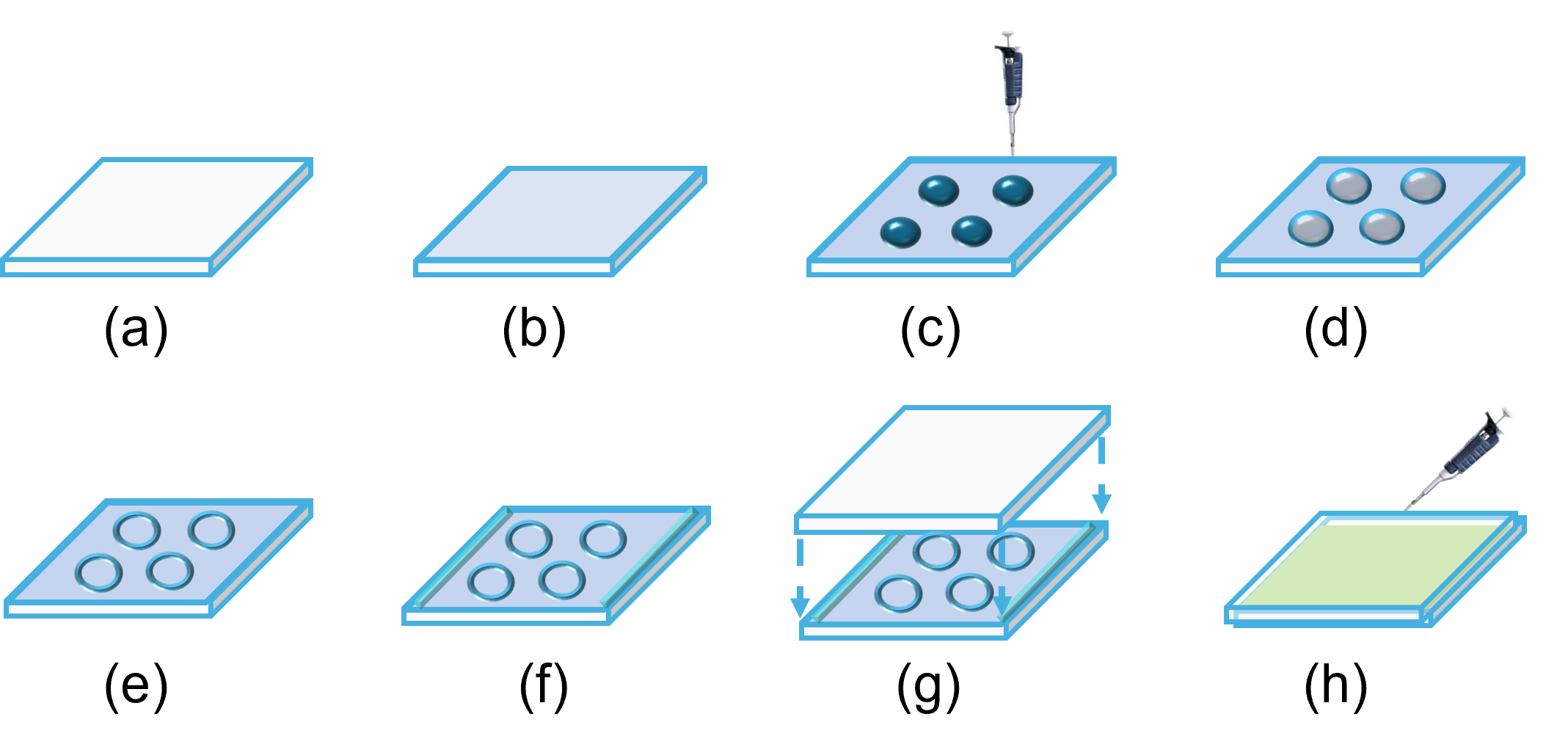


**Fig. S4.** Fabrication process of LC cells for BSA detection. (a) Cleaning the optical glass substrates. (b) Coating the cleaned substrates with 1-wt.% DMOAP for vertical alignment. (c) Dispensing 10 μL of BSA per spot onto the lower substrate to form a 2 × 2 array. (d) Drying the substrate at 30 °C. (e) Rinsing once with DIW to remove excess BSA and drying again. (f) Applying a mixture of AB glue and 15-μm spacers along the edges of the lower substrate. (g) Assembling the upper and lower substrates to form a LC cell. (h) Filling the cell with CLC by capillary action.

*Preparation of CLC cells for CA125 immunodetection*

For the CA125 immunodetection, the anti-CA125 antibody was immobilized on a lower DMOAP-modified substrate following the same procedure as that described in the previous subsection for BSA (Fig. S5(a)–(c)). Prior to immunoreaction, 40 μL of an aqueous solution of 1-M ETA was dispensed on the antibody-immobilized surface and covered with a glass cover slip to allow the blocking agent to react at room temperature for 30 minutes, in order to eliminate potential reactive sites on the glass substrate to react nonspecifically with CA125 (Fig. S5(d)). After the reaction, the glass cover slip was removed, and the glass substrate was rinsed with DIW to remove excess ETA (Fig. S5(e)). The CA125 solution was then applied following the same steps for the ETA reaction to complete the immunocomplex reaction (Fig. S5(f)–(g)). Finally, the CLC cell was assembled and filled with the 8CB/R5011 CLC as described previously for BSA detection (Fig. S5(h)–(j)).


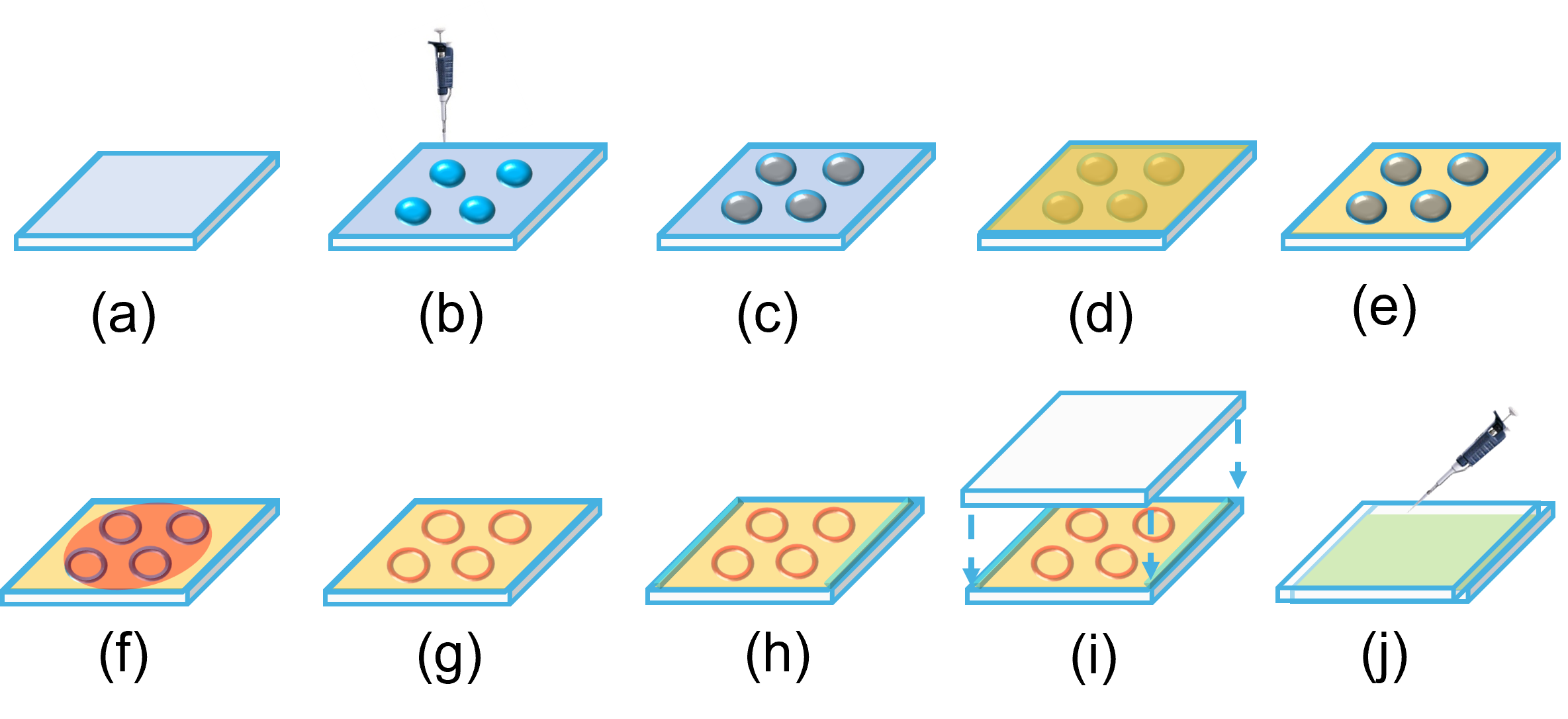


**Fig. S5.** Fabrication process of LC cells for CA125 immunodetection. (a) Coating the cleaned optical glass substrates with 1-wt.% DMOAP for vertical alignment. (b) Dispensing 10 μL of anti-CA125 antibodies per spot onto the lower substrate to form a 2 × 2 array. (c) Drying the substrate at 30 °C. (d) Dispensing 40 μL of 1 M ethanolamine (ETA) solution onto the center of the substrate and covering it with a cover glass, followed by reaction at room temperature for 30 minutes to block nonspecific binding. (e) Rinsing once with DIW to remove excess reagents, followed by drying. (f) Dispensing 40 μL of CA125 antigen onto the center of the substrate, covering it with a cover glass, and reacting for 30 minutes. (g) Rinsing once with DIW to remove unbound molecules, followed by drying. (h) Applying a mixture of AB glue and 15-μm spacers along the edges of the lower substrate. (i) Assembling the upper and lower substrates to form a LC cell. (j) Filling the cell with CLC by capillary action.

**S3. Simulations of birefringence-induced interference spectra and color generation in cross-polarized transmission through CLC textures**

Prior to color simulation, the relative intensity *I* (*λ*) of the halogen light source (Fig. S6(a)) within the POM was measured across the visible spectrum (400–700 nm) using a miniature spectrometer (PH2014-050-VNIR6, OtO Photonics Corp., Hsinchu, Taiwan). The refractive indices (*n*ₒ and *n*ₑ) of the host LC 8CB at 26.5 °C were determined at five specific wavelengths (486, 540, 589, 610, and 680 nm) by an Abbe refractometer (ATAGO DR-M4, ATAGO Corp., Tokyo, Japan) equipped with a FIRSTEK B401L recirculating cooling system and a Linkam T95-PE temperature controller to ensure stable measurement conditions (Fig. S6(b)). The resulting correlations between the refractive indices and wavelength were fitted using the extended Cauchy equation

$n_{e,o}(\lambda)=n_{e,o}^{\infty}+\frac{A_{e,o}}{\lambda^{2}}+\frac{B_{e,o}}{\lambda^{4}}$,

where *n*_e,o_ (*λ*) is the wavelength-dependent *n*_e_ or *n*_o_, $\text{n}_{\text{e,o}}^{\text{∞}}$ represents *n*_e_ or *n*_o_ as the wavelength approaches infinity, *A*_e,o_ and *B*_e,o_ are the Cauchy coefficients, and *λ* is the wavelength. The fitting parameters for *n*_e_ and *n*_o_ derived from Eq. (S3) are listed in Table S1. The coefficients of determination (*R*^2^) for the curve fittings were greater than 0.99 for both *n*_e_ and *n*_o_, indicating high reliability of the curves. The deduced dispersive functions enabled the subsequent simulation of the birefringence-induced interference spectrum and the prediction of the hue of the birefringent texture of the CLC cell serving as a biosensing platform.


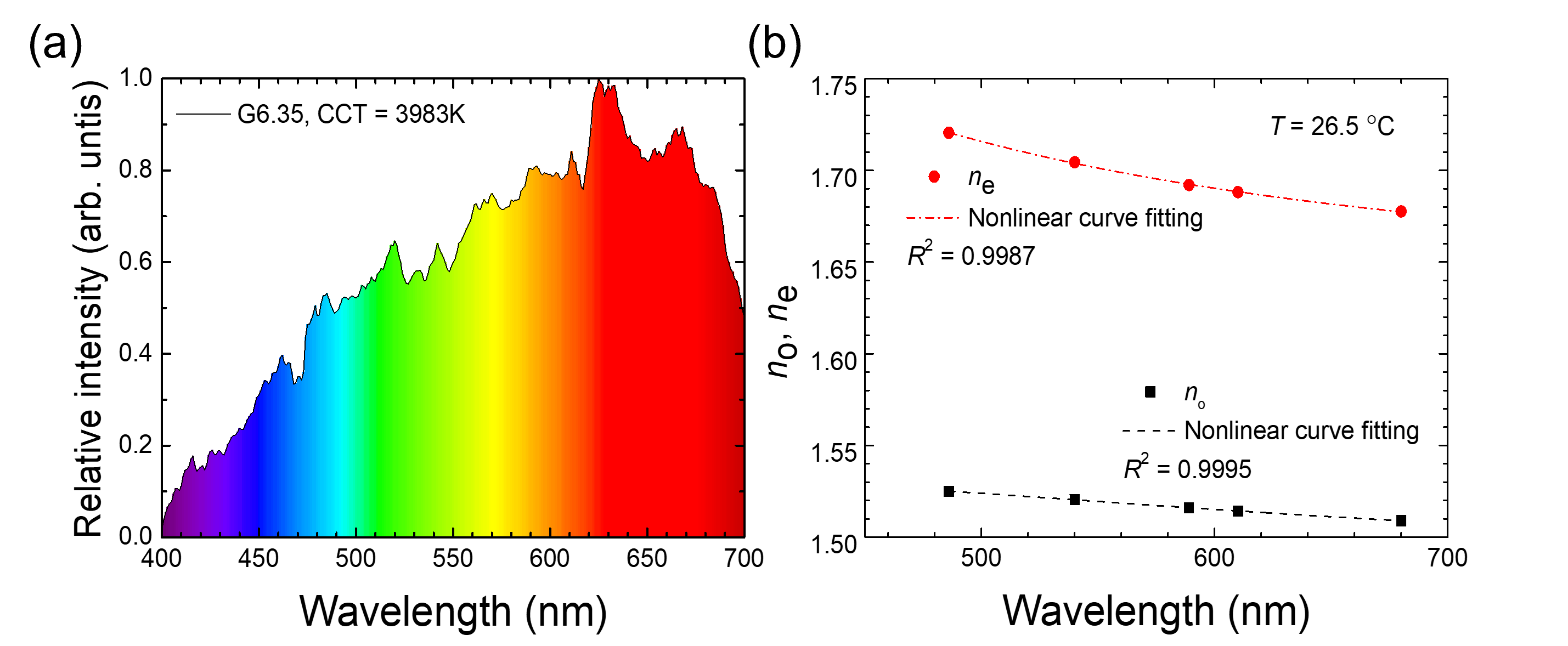


**Fig. S6.** Measurement of optical properties for the simulation of interference spectra and texture colors of the 8CB/R5011 CLC. (a) Relative intensity spectrum of the halogen light source in the OLYMPUS BX51-P polarizing optical microscope across the wavelength range of 400–700 nm. (b) Refractive indices *n*ₒ and *n*ₑ of 8CB, fitted to experimental data at the wavelengths of 486, 540, 589, 610, and 680 nm and **26.5 °C using the extended Cauchy equation over the visible spectrum (486–680 nm).**

**Table S1.** Fitting parameters and coefficients of determination for the ordinary and extraordinary refractive indices of 8CB, expressed using the extended Cauchy equation (Eq. (S3)).

| *n*_e_ | $n_{e}^{\infty}$ | *A*_e_ (μm^2^) | *B*_e_ (μm^4^) | *R*^2^ |
| --- | --- | --- | --- | --- |
|  | 1.63237 | 0.02077659849 | 1.77258 × 10^−5^ | 0.9987 |
| *n_o_* | $n_{o}^{\infty}$ | *A*_o_ (μm^2^) | *B*_o_ (μm^4^) | *R*^2^ |
|  | 1.47785 | 0.01775272451 | −1.56099 × 10^−3^ | 0.9995 |

Based on the obtained wavelength-dependent indices of refraction, the effective refractive index *n*_eff_ within the wavelength range of 400–700 nm was calculated for different LC tilt angles *θ* using the **effective refractive index equation** for a uniaxial crystal:

$n_{eff}=\frac{n_{e}n_{o}}{\sqrt{n_{o}^{2}\cos^{2}\theta+n_{e}^{2}\sin^{2}\theta}}$.

The value of *n*_eff_ was then substituted into the following equation to obtain the phase retardation *δ* at an arbitrary cell gap *d*:

$\delta=\frac{2\pi d}{\lambda}(n_{eff}-n_{o})$.

The cross-polarized transmittance *T*_⊥_ can then be derived by

,

where the azimuthal angle *ϕ* was set to be 45° for computational simplicity, considering only the phase retardation along the direction perpendicular to the substrate. By varying the value of *θ* to calculate *T*_⊥_ using Eqs. (S4)–(S6), the interference spectral profile of the 8CB/R5011 CLC at different cell gaps can be simulated to fit the measured *T*_⊥_-versus-wavelength curve at a designated tilt angle (Figs. 5(b), 5(c), 5(f), 5(g), S7(a), and S7(b)). Since the absolute value of the measured *T*_⊥_ was relatively small, the original data were normalized to 100% before simulating the profile of the interference spectrum in accordance with

$T_{norm}(\%)=\frac{T(\lambda\text{)}-T_{\min}}{T_{\max}-T_{\min}}\times100\%$,

where *T* (*λ*) and *T*_norm_ represent *T*_⊥_ before and after normalization, respectively, while *T*_max_ and *T*_min_ are the maximum and minimum values of *T*_⊥_, respectively, in the interference spectra. Finally, color simulation was performed by converting the spectral data of *T*_⊥_ as well as *I* (*λ*) in Fig. S6(a) into the CIE 1931 chromaticity coordinates (*x*, *y*) using

$$X(\lambda),\text{ }Y(\lambda),\text{ }Z(\lambda)=T_{\perp}\cdot x\text{(}\lambda\text{)},\text{ }y\text{(}\lambda\text{)},\text{ }z(\lambda)\cdot I(\lambda)$$

and

$x,\text{ }y=\sum_{i=400}^{700\text{ nm}} \frac{X_{i},\text{ }Y_{i}}{X_{i}+Y_{i}+Z_{i}}$.

These equations normalize the tristimulus values *X*, *Y*, and *Z* to project the color information onto the two-dimensional chromaticity diagram, where the third coordinate *z* is implicitly given by *z* = 1 – *x* − *y*. This representation effectively captures hue and saturation independent of luminance. Along with the experimental results, the simulated birefringence-induced interference spectra in cross-polarized transmission and the corresponding birefringent colors observed under the POM for CLC cells with thicknesses of 10 and 5.5 μm are depicted in Figs. S7(a)–(d) and (e) –(h), respectively.


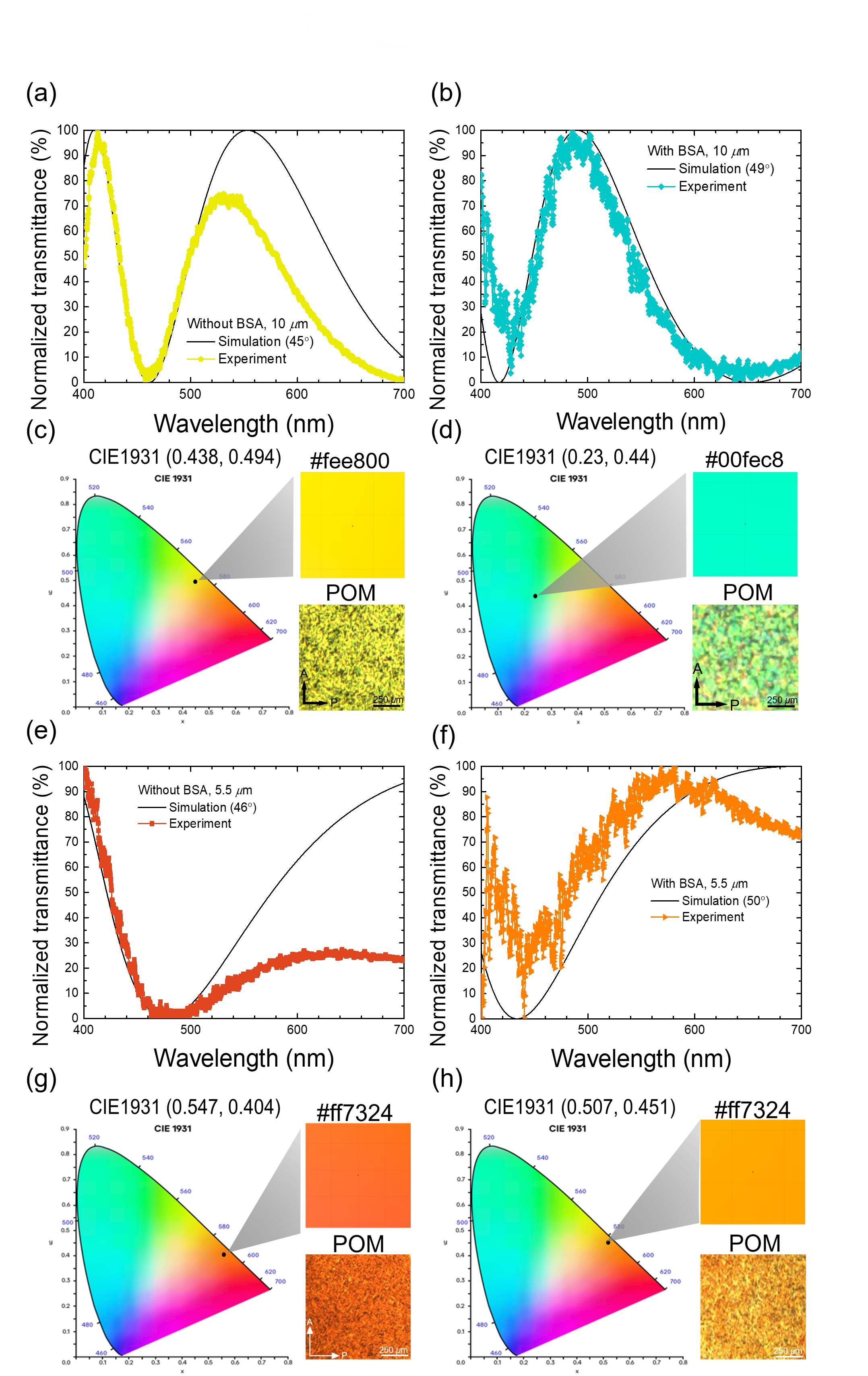


**Fig. S7.** Simulations of crossed-polarized transmission and color patterns in the POM textures of 8CB/R5011 CLCs. Simulated interference spectra at specific tilt angles fitted to normalized experimental data in the (a) absence and (b) presence of BSA at a cell gap of 10 μm. Comparison of texture colors predicted from the CIE 1931 chromaticity coordinates with observed POM images (c) without and (d) with BSA. (e)–(h) show simulated and experimental results for CLC cells with a 5.5-μm gap.

**S4. Comparison of the range of detection (ROD) and limit of detection (LOD) of protein assay techniques**

**Table S2** Comparison of the ROD and LOD of protein assay techniques.

| Protein assay techniques | ROD (g·mL^–1^) | LOD (g·mL^–1^) | References |
| --- | --- | --- | --- |
| Bradford protein assay (colorimetric) | 1–25 × 10^−6^ | 1.0 × 10^−6^ | Pierce™ Bradford Protein Assay Kit (ThermoFisher Scientifics) |
| Fluorescent dye-based protein assay | 10^−8^–10^−5^ | 1.0 × 10^−8^ | NanoOrange™ Protein Quantitation Kit (ThermoFisher Scientifics) |
| Fluorescent probe-based protein assay | 6.7× 10^−6^–10^−4^  (0.1–1.5 μM) | 2.6 × 10^−7^  (3.92 nM) | [[3](#_ENREF_3)] |
| Surface plasmon resonance probe-based protein assay | 10^−8^–10^−5^ | 5.7 × 10^−7^  (8.5 nM) | [[4](#_ENREF_4)] |
| Electrochemical protein assay | 10–25 × 10^−6^ | 1.0 × 10^−6^–1.0 × 10^−5^ | [[5](#_ENREF_5)] |
| CLC-based protein assay | 10^−12^–10^−5^ | 2.21 × 10⁻^11^  (image analysis) | This study |
|  | 10^−12^–10^−2^ | 1.23 × 10⁻^12^  (haze measurement) |  |
|  | 10^−12^–10^−2^ | 2.38 × 10⁻^12^  (based on Δ*T*_P_) |  |
|  | 10^−12^–10^−2^ | 1.35 × 10⁻^12^  (based on Δ*T*) |  |

**S5. Optimization of antibody concentration and demonstration of binding specificity**

As discussed in Section 3.4 about CLC-based quantitative CA125 immunoassay, the experimental results are illustrated in Fig. S8.


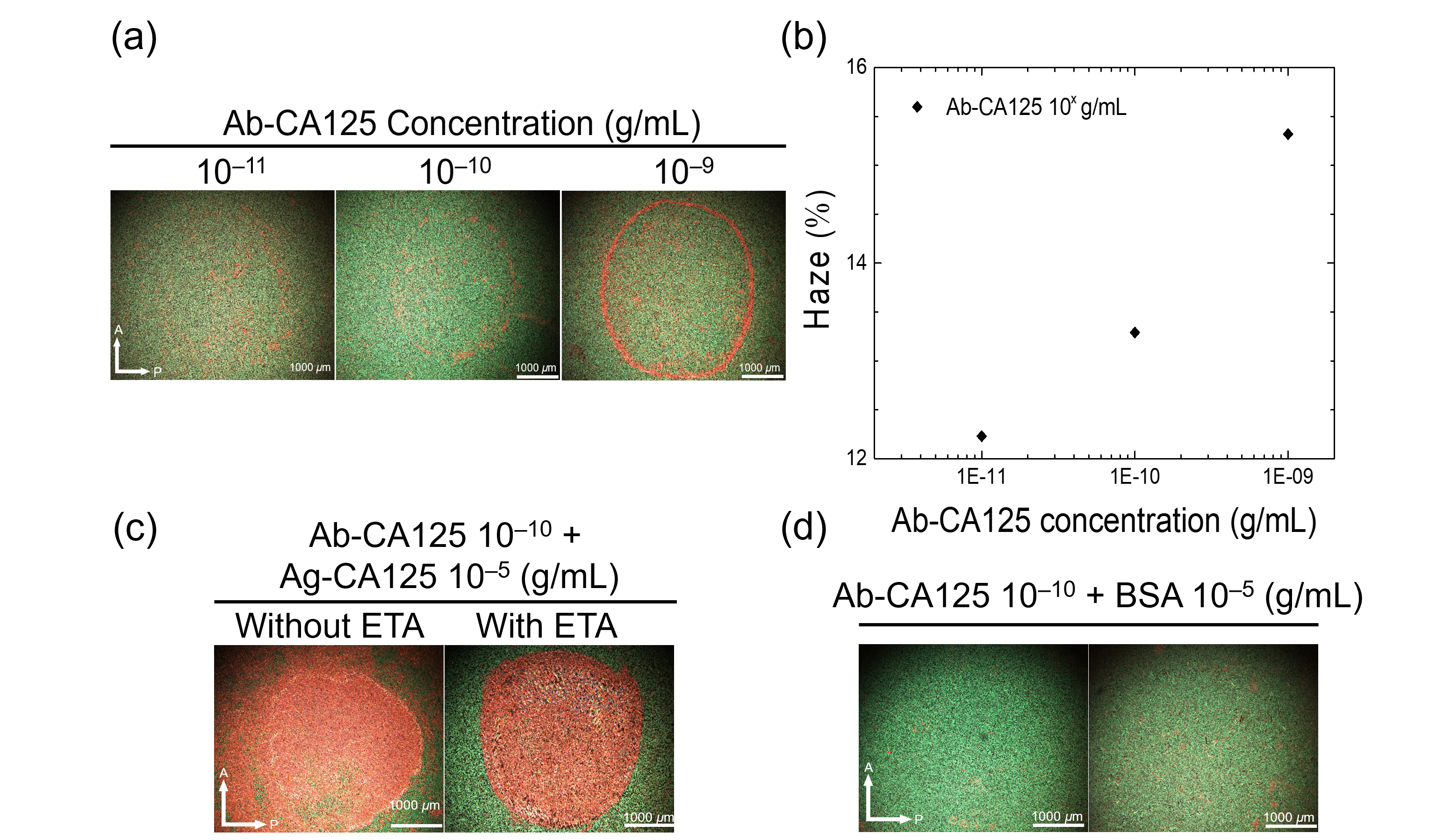


**Fig. S8.** Optimization of antibody concentration and demonstration of binding specificity. (a) Optical textures of the 8CB/R5011 CLC in the presence of three different immobilization concentrations of the anti-CA125 antibody (10⁻^11^, 10⁻^10^, and 10⁻^9^ g·mL⁻^1^). (b) Corresponding haze values of the CLC at the anti-CA125 antibody concentrations examined in (a). (c) The effect of ETA blocking on the optical textures of the 8CB/R5011 CLC, demonstrated by reacting 10⁻^10^-g·mL⁻^1^ immobilized anti-CA125 antibody with 10⁻^5^-g·mL⁻^1^ CA125. (d) Assessment of binding specificity of the CLC-based immunoassay by reacting 10⁻^10^-g·mL⁻^1^ immobilized anti-CA125 antibody with 10⁻^5^-g·mL⁻^1^ BSA.

**S6. Surface characterization by water contact angle (WCA) measurements**

To verify the immobilization of biomolecules, anti-CA125 antibodies were adsorbed on DMOAP-coated glass substrates, followed by immunoreaction with 1:100-diluted human serum spiked with various concentrations of CA125. Surface characterization was conducted through water contact angle (WCA) measurements using a 3 µL DI-water sessile drop. A shown in Fig. S9, the DMOAP-modified glass surface immobilized with 10⁻^10^-g·mL^–1^ anti-CA125 antibody was slightly hydrophilic, with an average WCA of *θ*(0) = 80.48 ± 1.80 °. Upon reaction with CA125, the WCA decreased in a concentration-dependent manner with increasing antigen concentration, reaching 60.05 ± 0.69 ° at 10⁻^5^-g·mL^–1^ CA125, indicating that formation of the antigen–antibody immunocomplex increases surface hydrophilicity. Surface characterization by WCA measurements provide direct evidence that biomolecular binding alters the surface condition of the glass substrate, thereby changing the interfacial interaction at the LC–glass interface and perturbing the CLC tilt angle.


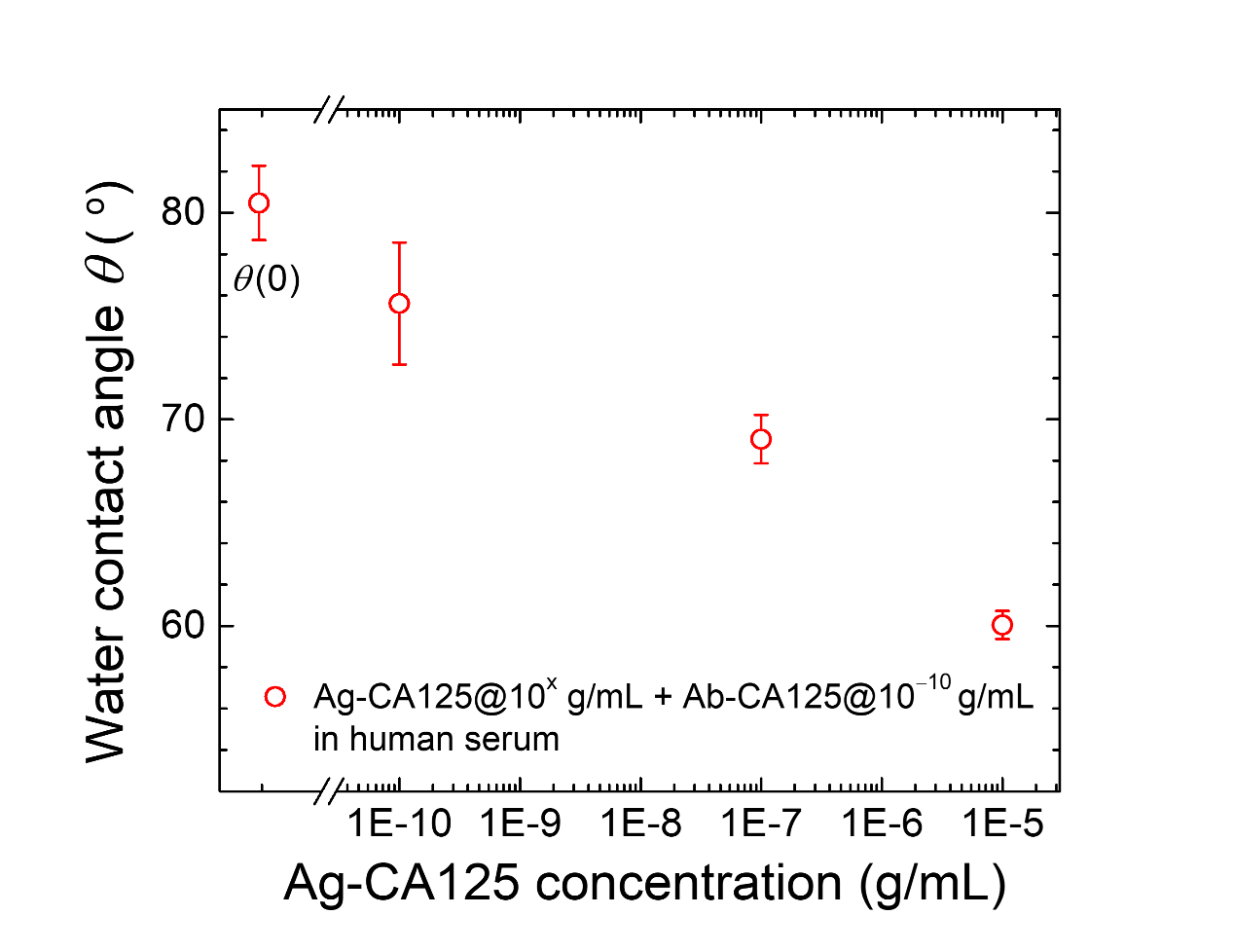


**Fig. S9.** Effect of CA125 immunocomplex formation on the water contact angle (WCA) of the DMOAP-coated glass surface. The anti-CA125 antibody was first immobilized on the DMOAP-coated glass surface, followed by interaction with diluted human serum spiked with various concentrations of CA125. WCA was then measured by a contact angle analyzer.

**References**

1. Ramanathan S, Gopinath SC, Hilmi Ismail Z, Subramaniam S. Nanodiamond conjugated SARS-CoV-2 spike protein: electrochemical impedance immunosensing on a gold microelectrode. Microchimica Acta. 2022;189(6):226.

2. Almeida LC, Frade T, Correia RD, Niu Y, Jin G, Correia JP, et al. Electrosynthesis of polydopamine-ethanolamine films for the development of immunosensing interfaces. Scientific reports. 2021;11(1):2237.

3. Mao J, Zhang Y, Zhang S, Song B. Turn-On Fluorescent Probe for BSA Detection Constructed by Supramolecular Assembly. Langmuir. 2024;40(10):5479-87. doi: 10.1021/acs.langmuir.4c00006.

4. Arcadio F, Zeni L, Perri C, D’Agostino G, Chiaretti G, Porto G, et al. Bovine Serum Albumin Protein Detection by a Removable SPR Chip Combined with a Specific MIP Receptor. Chemosensors. 2021;9(8):218.

5. Eksin E, Erdem A. Electrochemical detection of N-homocysteinylated BSA in the fetal bovine serum medium. RSC Advances. 2015;5(7):4774-9. doi: 10.1039/C4RA13303J.
